# A *Colletotrichum graminicola* mutant deficient in the establishment of biotrophy reveals early transcriptional events in the maize anthracnose disease interaction

**DOI:** 10.1186/s12864-016-2546-0

**Published:** 2016-03-08

**Authors:** Maria F. Torres, Noushin Ghaffari, Ester A. S. Buiate, Neil Moore, Scott Schwartz, Charles D. Johnson, Lisa J. Vaillancourt

**Affiliations:** Department of Plant Pathology, University of Kentucky, 201F Plant Science Building, 1405 Veterans Drive, Lexington, KY 40546-0312 USA; AgriLife Genomics and Bioinformatics, Texas A&M AgriLife Research, Texas A&M University, College Station, TX 77845 USA; Department of Computer Science, University of Kentucky, Davis Marksbury Building, 328 Rose Street, Lexington, KY 40506-0633 USA; Present Address: Functional Genomics Laboratory, Weill Cornell Medical College, Cornell University, Qatar Foundation – Education City, Doha, Qatar; Present Address: Monsanto Company Brazil, Uberlândia, Minas Gerais Brazil; Present Address: Department of Integrative Biology, University of Texas, Austin, TX 78712 USA

**Keywords:** RNA-Seq, Plant disease, Fungal development, Transcriptional profiling, Biotrophic development

## Abstract

**Background:**

*Colletotrichum graminicola* is a hemibiotrophic fungal pathogen that causes maize anthracnose disease. It progresses through three recognizable phases of pathogenic development *in planta*: melanized appressoria on the host surface prior to penetration; biotrophy, characterized by intracellular colonization of living host cells; and necrotrophy, characterized by host cell death and symptom development. A “Mixed Effects” Generalized Linear Model (GLM) was developed and applied to an existing Illumina transcriptome dataset, substantially increasing the statistical power of the analysis of *C. graminicola* gene expression during infection and colonization. Additionally, the *in planta* transcriptome of the wild-type was compared with that of a mutant strain impaired in the establishment of biotrophy, allowing detailed dissection of events occurring specifically during penetration, and during early versus late biotrophy.

**Results:**

More than 2000 fungal genes were differentially transcribed during appressorial maturation, penetration, and colonization. Secreted proteins, secondary metabolism genes, and membrane receptors were over-represented among the differentially expressed genes, suggesting that the fungus engages in an intimate and dynamic conversation with the host, beginning prior to penetration. This communication process probably involves reception of plant signals triggering subsequent developmental progress in the fungus, as well as production of signals that induce responses in the host. Later phases of biotrophy were more similar to necrotrophy, with increased production of secreted proteases, inducers of plant cell death, hydrolases, and membrane bound transporters for the uptake and egress of potential toxins, signals, and nutrients.

**Conclusions:**

This approach revealed, in unprecedented detail, fungal genes specifically expressed during critical phases of host penetration and biotrophic establishment. Many encoded secreted proteins, secondary metabolism enzymes, and receptors that may play roles in host-pathogen communication necessary to promote susceptibility, and thus may provide targets for chemical or biological controls to manage this important disease. The differentially expressed genes could be used as ‘landmarks’ to more accurately identify developmental progress in compatible versus incompatible interactions involving genetic variants of both host and pathogen.

**Electronic supplementary material:**

The online version of this article (doi:10.1186/s12864-016-2546-0) contains supplementary material, which is available to authorized users.

## Background

The fungus *Colletotrichum graminicola* Ces. Wils. is the causal agent of anthracnose leaf blight and anthracnose stalk rot diseases of maize [1]. ASR is one of the most economically important maize diseases, and is estimated to cause billions of dollars in losses annually in the United States [2]. A better understanding of the molecular mechanisms of *C. graminicola* pathogenicity to maize leaves and stalks might lead to improved methods for disease management.

Most plant pathogens can be classified into one of two groups based on their feeding strategies. Necrotrophic pathogens induce host cell death in advance of their growth, and then feed on the dead plant tissues [3]. Biotrophic pathogens, in contrast, invade living host cells and reprogram them, using a variety of molecular signals, to divert nutrients for their own use [3, 4]. Some pathogens, including *C. graminicola*, have a hemibiotrophic lifestyle that appears to be intermediate between these two extremes. After mechanically penetrating the plant epidermis via a melanized appressorium, *C. graminicola* grows initially as a biotroph, producing thick primary hyphae that invade living host cells, and are separated from the host cytoplasm by a membrane. Later, it switches to necrotrophic growth, producing thinner secondary hyphae that colonize dead cells and are no longer surrounded by a membrane [5–11]. Host tissue collapse and necrotic symptoms occur only during the necrotrophic phase of development [1, 4, 11–14].

The genome of *C. graminicola* includes a large number of genes that are associated with the production of secondary metabolites (SM) [15], a feature that is reminiscent of many necrotrophic plant pathogens that secrete phytotoxic compounds to kill plant cells ahead of colonization [16, 17]. However, *C. graminicola* has not been observed to kill host cells in advance, and each new cell is invaded while it is still alive [11]. This behavior is more like that of biotrophic pathogens, which suppress host cell death and defense pathways by producing a wide variety of secreted proteins known as “effectors” [18–21]. Fungal effectors are typically characterized as small, secreted proteins (SSPs), usually induced *in planta*, which function to facilitate pathogen colonization [15, 22–25]. Fungal SSP effectors are often enriched in cysteine residues (SSP-CRs): cysteine-rich proteins may be more stable and resistant to plant proteases during infection [26]. The *C. graminicola* genome encodes hundreds of putative SSP and SSP-CR effectors [15, 27]. However, plant defense responses are reportedly activated during the earliest stages of infection of maize leaves, when the fungus still appears to be growing biotrophically [28] and cells die rapidly after biotrophic invasion [11], showing that biotrophy in *C. graminicola* differs fundamentally from that in true biotrophic pathogens.

Biotrophic invasion by *C. graminicola* continues at the edges of the expanding lesion, even as the pathogen switches to necrotrophic growth in the center [11]. Thus biotrophy and necrotrophy coexist in *C. graminicola* lesions. A similar growth pattern has been observed in the closely related sorghum anthracnose pathogen, *C. sublineola* [29, 30]. Contrasting with these two species, the biotrophic hyphae of *C. higginsianum*, a pathogen of *Arabidopsis thaliana* and other Brassicaceae, persist in only one cell before making a complete switch to necrotrophy for invasion of subsequent cells [15]. Analysis of differential gene expression patterns in biotrophic hyphae of *C. higginsianum* suggested that they functioned primarily to produce SSPs and SMs to support the establishment of biotrophy and the subsequent switch to necrotrophy [15, 31]. A preliminary description of the transcriptome of *C. graminicola* during the biotrophic phase of development *in planta* was presented previously [15]. However, in contrast with *C. higginsianum*, it was not possible to obtain detailed information about differential patterns of gene expression in the biotrophic hyphae of *C. graminicola*, probably because of the asynchronous nature of the infection.

In the present work, we have continued our analysis of our *in planta* RNA-Seq transcriptome data from *C. graminicola* by utilizing improved methods that have allowed us to map more reads to the fungal genome, and by applying a more rigorous statistical analysis of differential gene expression during pre-penetration, biotrophic, and necrotrophic phases of development. We developed a “Mixed Effects” Generalized Linear Model (GLM), using the edgeR [32] package of Bioconductor [33]. The Bioconductor is an open source project that includes tools for high-throughput genomic data analysis. Bioconductor packages are based on the R language, and support object-oriented frameworks, visualization tools, and vignette documents. R is the programming language of choice for statistical and computing applications [34], and its data structure and codes are used by Bioconductor packages. We chose the edgeR package due to its well-known capability in RNA-Seq analysis, especially its powerful GLM-based differential expression analysis [35].

We applied the same methods to compare the *in planta* transcriptome of the pathogenic wild-type strain of *C. graminicola* (WT) with that of a non-pathogenic mutant strain (MT) that is impaired specifically during early biotrophic development [11, 36]. This strain has an insertional mutation in a gene (*Cpr1*) that is predicted to encode one subunit of the signal peptidase complex [36, 37]. The MT germinates and produces appressoria normally on maize leaves and leaf sheaths, but penetration is delayed, and once inside it fails to progress beyond the first biotrophically colonized host cell [11]. Comparison of the MT with the WT allowed us to characterize the transcriptional activity of *C. graminicola* during penetration, and during early versus later stages of biotrophy, in more detail than previously possible in this species. This analysis enabled us to identify genes that are expressed specifically at these critical points in disease establishment, including genes encoding putative SSP effectors, SM enzymes, membrane receptors, and transcription factors. These genes can be subjected to more detailed functional analyses in the future in order to understand their roles in biotrophic development. The products of some of these genes may provide novel targets for improved control of this important disease.

## Results and discussion

### Overview of the RNA sequencing results and statistical analysis

A total of ~3.5 × 10^8^ Illumina sequencing reads were obtained from samples consisting of the WT and MT strains at three different stages of *in planta* development (treatments): pre-penetration appressoria (AP); biotrophy (BT); and necrotrophy (NT: WT only) (Additional file 1: Figure S1; Additional file 2: Table S1). Most of the reads were derived from the plant transcriptome (data not included), but a total of 2.2 × 10^7^ reads (6.2 %) could be mapped to the fungal genome (Genbank accession GSE71919) (Additional file 2: Table S1). The percentage of mapped WT reads in the current study was increased to 9.5 %, compared with only 5.6 % reported for the previous analysis [15] (Genbank accession PRJNA151285) (Additional file 2: Table S1). It should be noted that the previous study did not include the MT strain.

**Fig. 1:**
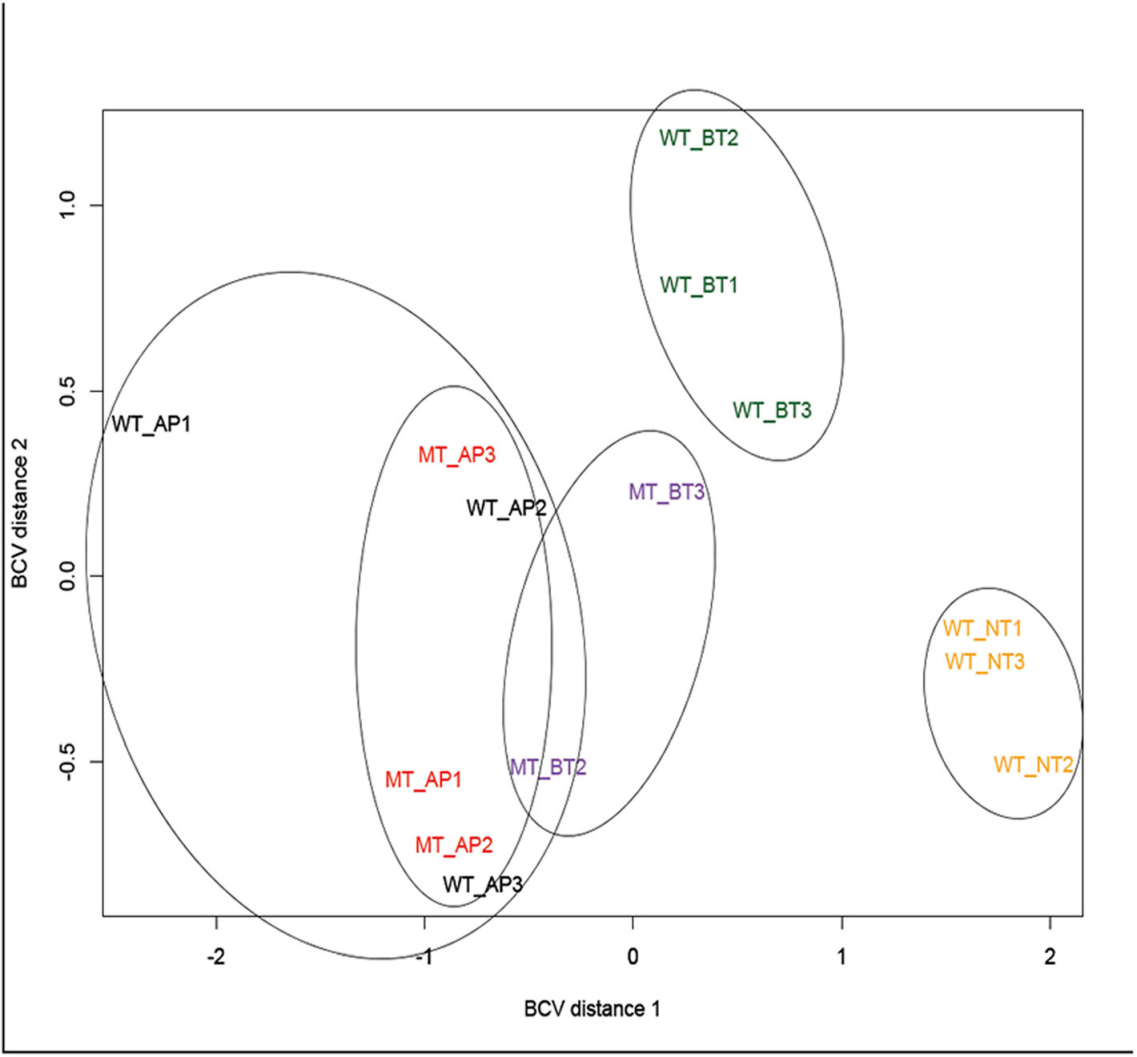
EdgeR MDS plots of three biological replicates each of the WT appressorial phase (WT_AP1, WT_AP2, WT_AP3); the WT biotrophic phase (WT_BT1, WT_BT2, WT_BT3); the WT necrotrophic phase (WT_NT1, WT_NT2, WT_NT3); and the MT appressorial phase (MT_AP1, MT_AP2, MT_AP3): and two biological replicates of the MT biotrophic phase (MT_BT2, MT_BT3)

More than 95 % of the annotated *C. graminicola* genes were expressed at some point during infection. EdgeR Multidimensional Scaling (MDS) plots showing the spatial location of data (treatment) clusters indicated that the WT appressorial (WTAP), MT appressorial (MTAP), and MT biotrophic (MTBT) phases were similar to one another, while the WT biotrophic (WTBT) and the WT necrotrophic (WTNT) phases were distinct (Fig. 1).

### Identification and validation of differentially expressed genes

Counts Per Million (CPM) is the default data filtering method of the edgeR package. As an alternative to the CPM, we applied our own filtering method, mainly because CPM did not account adequately for our sample replicates. In the first round of our filtering method, we chose genes with a total of at least 20 reads across all replicates of at least one treatment. In the next step, genes that had at least 3 treatments with a total of at least 15 reads across all replicates were selected. We determined that the lists of filtered genes generated by our method and by the default CPM method were more than 96 % identical, and also in each comparison there was more than 93 % agreement. However our method avoided screening out of genes that actually had sufficient read depth to be included, when replicates were taken into account.

Eighty-four percent of the *C. graminicola* predicted genes (10,028/12,006) had sufficient read depth to include after filtering. Differential expression analysis of these genes via edgeR identified 2412 statistically significant differentially expressed genes across the various comparisons (FDR ≤0.05, Log2 fold change >2) (Additional file 2: Table S2). This is compared with 2619 differentially expressed genes identified in our previous study [15]. However, the former analysis included only P value and Log2 fold change. Application of the more rigorous FDR criterion to the previous dataset reduces the number of statistically significant differentially expressed genes to 1855.

The RNA-Seq data for fourteen differentially expressed genes were validated by using quantitative real-time reverse-transcription polymerase chain reaction (qRT-PCR). Transcript log2 fold-changes (AP vs BT, BT vs NT, AP vs NT) measured by RNA-Seq and qRT-PCR had a positive correlation of *R*^2^ = 0.8604, and a simple linear model slope of y = 1.036 (Additional file 1: Figure S2), supporting the reliability of the RNA-Seq data.

### One in five fungal genes is differentially expressed during development of the WT *in planta*

**Fig. 2:**
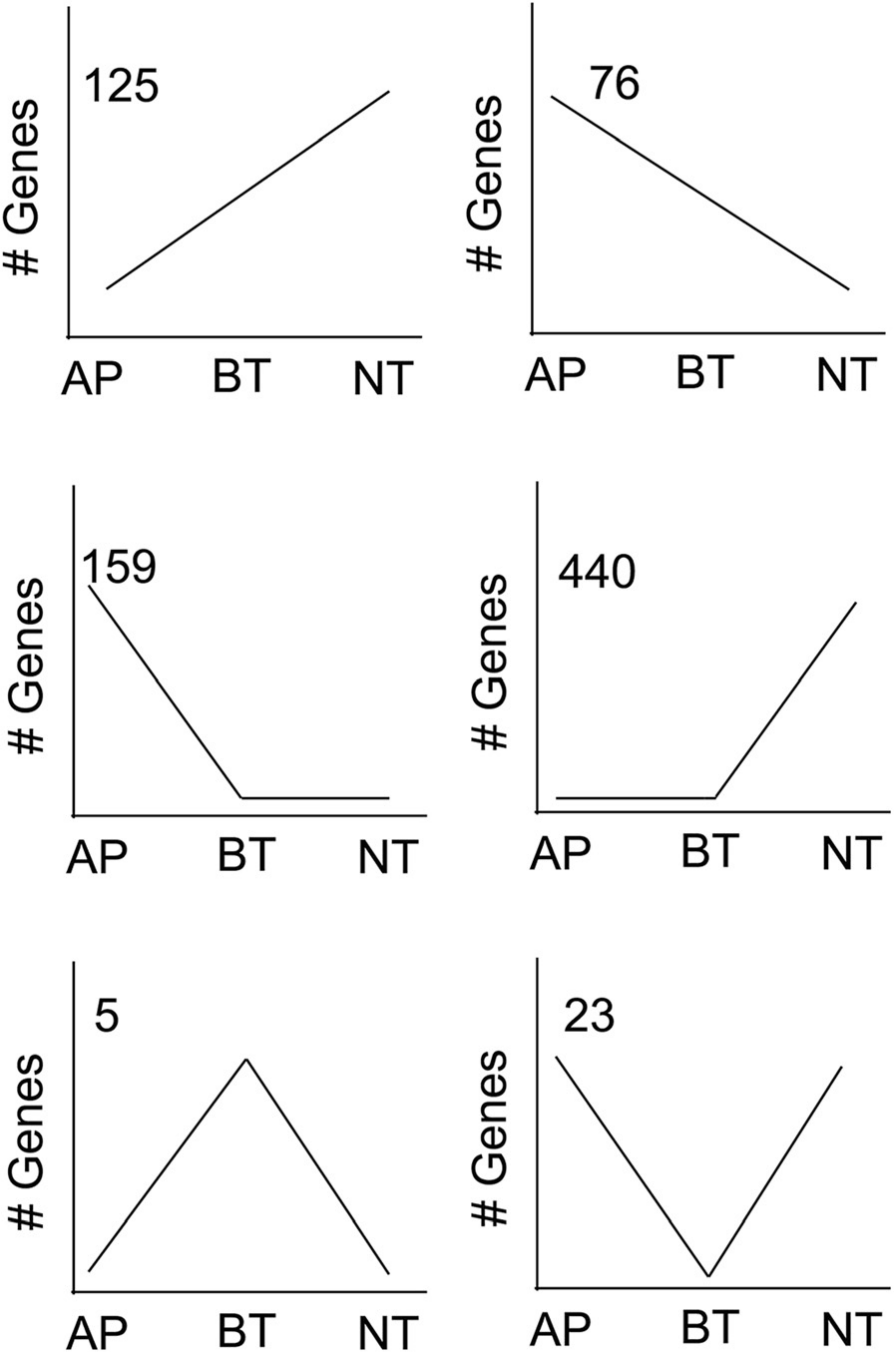
Six patterns of relative expression among the differentially expressed genes during colonization of maize by the WT. The number of genes with each pattern is given in the top left corner of each graph. The patterns are not drawn to scale, and are intended to represent the relative amounts of transcript at each developmental stage

About 20 % of the fungal genes were differentially transcribed across the processes of appressorial maturation, penetration, and colonization in the WT *in planta* (Table 1, Additional file 2: Table S2). These genes could be grouped into six classes, each with a different pattern of expression (Fig. 2). One group of 125 genes increased progressively in expression from the AP through the BT to the NT phases, while another group of 76 genes had the opposite pattern, progressively decreasing in expression during the three sequential developmental phases (Fig. 2, Additional file 2: Table S2). More than 100 “early” genes were more highly expressed specifically in AP, and another much larger group of “late” genes, was increased in expression specifically during NT (Fig. 2, Additional file 2: Table S3). There were only five genes that were more highly expressed specifically in BT, while 23 were decreased in expression specifically during that phase (Fig. 2, Table 2).

**Table 1:** Summary of the number of genes with significantly different expression (FDR ≤0.05, Log2 fold change >2) between different fungal stage comparisons

Comparison	Differentially expressed	Higher expression^*^	Lower expression^*^
WTAP vs. WTBT	760	293 (WTAP_WTBT_up)	467 (WTAP_WTBT_dn)
WTAP vs. WTNT	1935	728 (WTAP_WTNT_up)	1207 (WTAP_WTNT_dn)
WTBT vs. WTNT	992	312 (WTBT_WTNT_up)	680 (WTBT_WTNT_dn)
MTAP vs. MTBT	20	0 (MTAP_MTBT_up)	20 (MTAP_MTBT_dn)
WTAP vs. MTAP	218	74 (WTAP_MTAP_up)	144 (WTAP_MTAP_dn)
WTBT vs. MTBT	714	192 (WTBT_MTBT_up)	522 (WTBT_MTBT_dn)

^*^ Higher and lower expression refers in each case to the first term in the comparison relative to the second

**Table 2:** Genes that are differentially expressed specifically during the WT biotrophic phase of development

Gene ID	log2 fold-change*	Pfam	Description	PHI database hit
GLRG_00351	−2.30/2.63	PF00135	Carboxylesterase family	*LIP1 Botrytis cinerea*
GLRG_00814	−2.61/2.53	PF12296	Hydrophobic surface binding protein A	
GLRG_01112	−5.06/4.32	PF00132	Bacterial transferase hexapeptide (six repeats) (probable transcription factor)	GzZC087 *Gibberella zeae*
GLRG_01787	−5.40/4.80	PF06609	Fungal trichothecene efflux pump (TRI12)	*TRI12 Fusarium sporotrichoides* (toxin associated)
GLRG_01790	−2.80/2.93	PF00501	AMP-binding enzyme	*AKT1 Alternaria alternate* (toxin associated)
**GLRG_01888**	3.49/-3.44	PF02492	CobW/HypB/UreG, nucleotide binding domain	
GLRG_01911	−2.04/2.87	PF04082	Fungal specific transcription factor domain	GzZC297 *Gibberella zeae*
**GLRG_02025**	2.34/-2.01	PF06985	Heterokaryon incompatibility protein (HET)	
GLRG_02289	−3.11/5.00	PF00246	Zinc carboxypeptidase	
GLRG_02897	−3.55/2.04	PF00067	Cytochrome P450	Related to O-methylsterigmatocystin oxidoreductase *Gibberella ze*ae (toxin associated)
GLRG_03377	−2.96/4.18		Predicted nuclear conserved hypothetical protein	
**GLRG_03389**	2.13/-2.97	PF13434	L-lysine-6-monooxygenase (NADPH-requiring)	
GLRG_03699	−4.21/3.03		Predicted cytosolic conserved hypothetical protein	
GLRG_04803	−3.73/3.47	PF07690	Major Facilitator Superfamily	GzCCHC002 *Gibberella zeae*
GLRG_06419	−4.45/3.42	PF01557	Fumarylacetoacetate (FAA) hydrolase family	
GLRG_07902	−5.60/5.70	PF12697	Alpha/beta hydrolase family	
**GLRG_08904**	7.13/-2.34	PF05730	CFEM domain	*PTH11 Magnaporthe oryzae*
GLRG_09112	−4.76/7.81	PF00004	ATPase family associated with various cellular activities (AAA)	
**GLRG_09541**	2.77/-2.52	PF07690	Major Facilitator Superfamily	MGG_10702 *Magnaporthe oryza*e
GLRG_09602	−3.40/9.29		Small secreted protein putative effector, similar to MGG_02647	
GLRG_09749	−2.51/5.04	PF13738	Pyridine nucleotide disulphide oxidoreductase	*DEP4 Alternaria brassicicola* (toxin associated)
GLRG_10073	−3.14/4.44	PF01822	Secreted WSC domain protein	
GLRG_10235	−3.24/3.21	PF01177	Asp/Glu/Hydantoin racemase	
GLRG_10715	−5.44/5.55	PF00753	Metallo-beta-lactamase superfamily	
GLRG_10836	−4.65/5.48	PF00916	Sulfate transporter family	
GLRG_11179	−5.25/5.71		Predicted mitochondrial protein, unique to *C. graminicola*	
GLRG_11184	−4.87/4.83	PF00106	Short chain dehydrogenase	*MFP1 Magnaporthe oryzae*
GLRG_11827	−2.68/3.08		Predicted nuclear protein, unique to *C. graminicola*	

^*^First number is log2 fold-change from AP to BT, second is from BT to NT

Genes that are more highly expressed specifically during biotrophy are highlighted in bold, while the rest are reduced in expression specifically during biotrophy

### Relatively few genes were differentially expressed in the MT

For the MT, only two phases of development occurred in leaf sheaths (AP and BT) (Additional file 1: Figure S1). Only 20 genes were differentially expressed between these two phases in the MT, all of which were more highly expressed during BT (Tables 1 and 3). This result is consistent with our previous observations that the MT biotrophic phase is arrested very early in its development [11].

**Table 3:** Genes that are differentially expressed during the transition from AP to BT in the mutant

Gene ID	log2 fold-change	Pfam	Description	PHI Hit	Category
GLRG_00048	5.18	PF13489	Methyltransferase domain	*ChLae1 Cochliobolus heterostrophus*	
GLRG_00795	8.16	PF00933	Glycosyl hydrolase family 3 N terminal domain	*TOM1 Septoria lycopersici*	Cazyme (GH3)
GLRG_01187	5.15	PF00067	Cytochrome P450	*BcBOT1* (related *CND5*) *Botrytis cinerea*	
GLRG_01737	3.98	PF13848	Thioredoxin-like domain		
GLRG_03450	7.20	PF04628	Sedlin, N-terminal conserved regions		
GLRG_03485	8.14		Predicted small mitochondrial protein, unique to *C. graminicola*		
GLRG_04091	7.62	PF04389	Secreted aminopeptidase M28A		Secreted protease
GLRG_04432	5.06		Predicted plasma membrane protein	*PTH11 Magnaporthe oryzae*	
GLRG_05524	5.32	PF00457	Glycosyl hydrolases family 11	*XYL2 Cochliobolous carbonum*	Cazyme (GH11)
GLRG_06219	4.72	PF00246	Secreted Zinc carboxypeptidase M14A		Secreted protease
GLRG_06274	3.87	PF03443	Glycosyl hydrolase family 61	GzOB021 *Gibberella zeae*	Cazyme (AA9)
GLRG_06286	3.67	PF05572	Pregnancy-associated plasma protein-A: secreted metalloprotease M43B	*MEP1 Coccidioides posadasii*	Secreted protease
GLRG_07653	4.99		Extracellular conserved hypothetical protein		
GLRG_08966	8.40	PF00331	Glycosyl hydrolase family 10	Endo-1,4-beta-xylanase *M. oryzae* PHI_2208	Cazyme (GH10-CBM1)
GLRG_08975	3.55	PF01738	Dienelactone hydrolase family		Conserved SSP
GLRG_09712	5.18	PF12695	Alpha/beta hydrolase family		Cazyme, (CE1). SM Cluster 22
GLRG_09714	7.40	PF00107	Zinc binding dehydrogenase		SM Cluster 22
GLRG_09807	7.70	PF00734	Fungal Cellulose Binding Domain	*FAED1 Gibberella zeae*	Cazyme, (CE1-CBM1)
GLRG_11440	4.59				Conserved SSP, cysteine rich
GLRG_11800	2.80	PF00330	Aconitase family (aconitase hydratase)	*LYSF Aspergillus fumigatus*	

All are more highly expressed during BT compared with AP. SM = secondary metabolism, SSP = small secreted protein

### The *Cpr1* gene is not differentially expressed *in planta* in the MT or WT strains

The MT strain has an insertion of foreign DNA into the 3′ untranslated region of the *Cpr1* gene (GLRG_04964), which is predicted to encode a non-catalytic component of the signal peptidase [36]. Analysis of the RNA-Seq data indicated that *Cpr1* was expressed at similar levels in the WT and in the MT *in planta* across all phases of development.

**Fig. 3:**
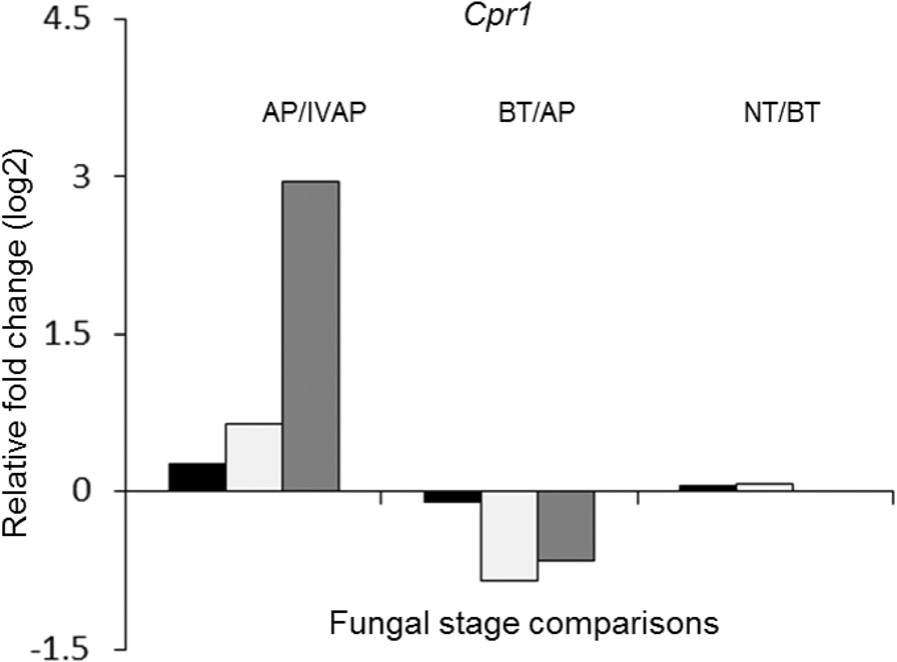
Relative expression of *Cpr1* during different stages of fungal infection in WT (black bars), *Cpr1-C* (white bars) and *cpr1* mutant (dark gray bars), measured by quantitative RT-PCR. Expression values are shown as fold changes relative to expression in other fungal stages. IVAP = in vitro appressoria. AP = appressoria. BT = biotrophic stage. NT = necrotrophic stage

The lack of differential expression of *Cpr1 in planta* was confirmed by using qRT-PCR. RNA samples isolated from mature appressoria induced on an artificial surface (IV-AP), and leaf sheaths inoculated with the complemented MT strain (*Cpr1*-C) [36], were also included in this analysis as controls. The qRT-PCR data confirmed that there was less than a 2-fold variation in the expression of this gene in all three strains during each transition *in planta* (Fig. 3). Thus, *Cpr1* appears to be regulated normally at the transcriptional level in the MT strain *in planta*. In contrast, expression of *Cpr1* in the IV-AP of the MT strain was reduced 8-fold in comparison with MT appressoria produced on the host plant surface (Fig. 3), whereas expression levels were similar for WT and *Cpr1*-C appressoria produced in vitro versus *in planta*. We showed previously that *Cpr1* transcript levels were substantially reduced in MT mycelia growing in rich medium when compared with WT mycelia growing in the same medium [36]. These findings suggest that transcript levels of *Cpr1* are responsive to plant signals, and also that the nonpathogenic phenotype of the MT may relate to post-transcriptional or post-translational regulation of *Cpr1*.

### Membrane-protein and secreted-protein genes are over-represented among the differentially expressed genes

**Fig. 4:**
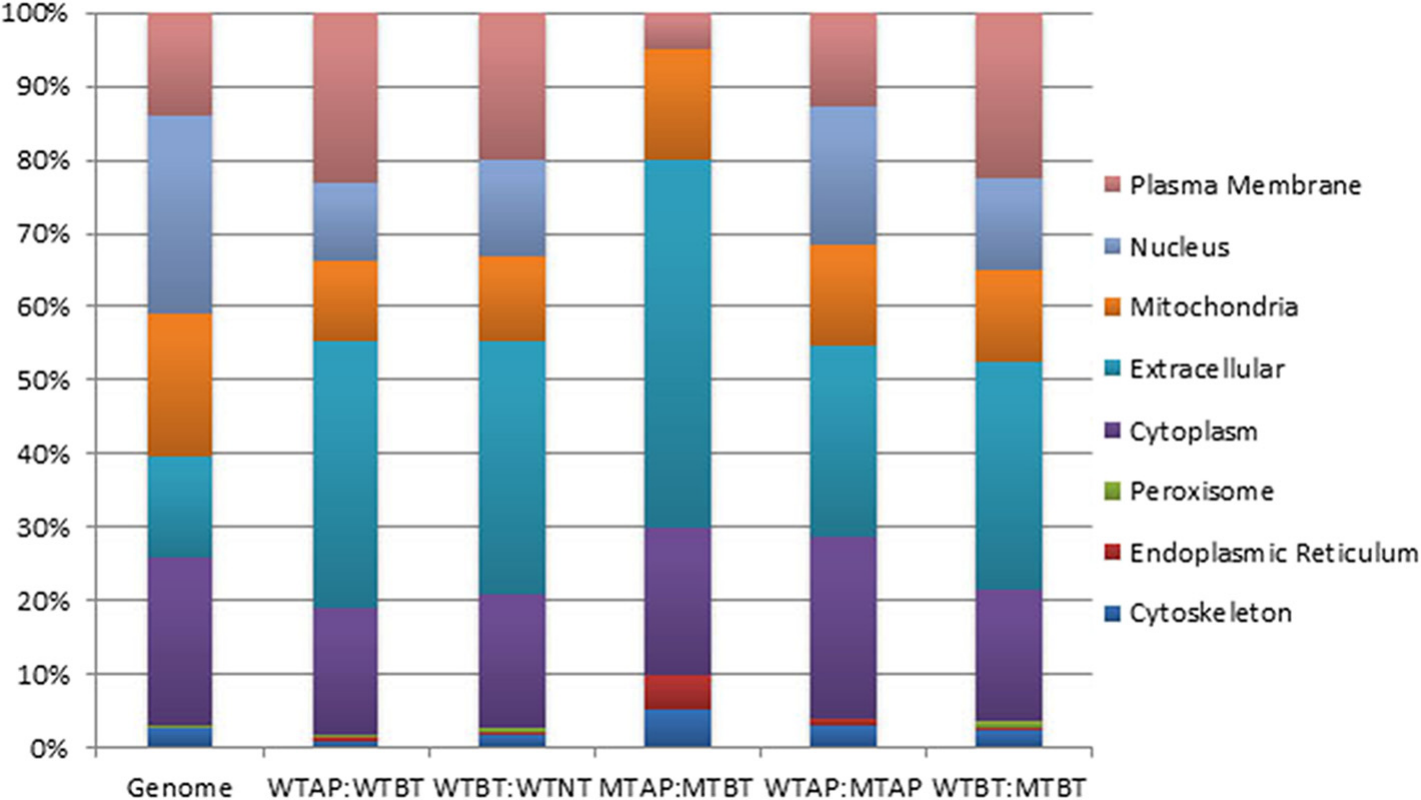
Predicted cellular localizations for putative proteins encoded by the *C. graminicola* genome, and by differentially expressed genes in different comparisons. X axis shows the percentage of predicted proteins in each category

Membrane receptors and secreted proteins are likely to play roles in communication between the plant and the pathogen, and thus to be especially important for the successful colonization of maize by *C. graminicola*. Almost 14 % of the genes encoded by *C. graminicola* are expected to encode secreted proteins, and a similar percentage encodes proteins that are predicted to localize to the plasma membrane (Fig. 4). In contrast, about a third of the differentially expressed genes are predicted to encode secreted proteins, and approximately 20 % encode plasma membrane proteins, across the different comparisons (Fig. 4, Additional file 2: Table S4). These proteins are enriched in SSPs, secreted proteases, carbohydrate-active enzymes (CAZymes), transporters, and other categories likely to be involved in pathogenicity (Table 4). Each developmental stage (AP, BT, and NT) was characterized by the expression of a distinct subset of these genes, presumably with specific functions related to fungal colonization and survival at each phase. Each developmental transition was also characterized by the up- or down-regulation of a unique set of transcription factors, which may control the differential expression of the other genes.

**Table 4:** Relative representation of differentially-expressed genes encoding potential pathogenicity-associated categories of proteins in different comparisons

Comparison (number of genes)	SSP	PHI-base	Secreted protease	Cazyme	Transporter	Secondary metabolite
Total Genome (12,006)	687 (6 %)	3224 (27 %)	110 (1 %)	704 (6 %)	1476 (12 %)	300 (3 %)
WT Early Genes (159)	24 (15 %)	55 (35 %)	2 (1 %)	14 (9 %)	13 (8 %)	14 (9 %)
WT Late Genes (440)	42 (10 %)	144 (33 %)	6 (1 %)	77 (18 %)	48 (11 %)	26 (6 %)
WTBT_up (5)	0	2 (40 %)	0	0	2 (40 %)	0
WTBT_dn (23)	2 (9 %)	9 (39 %)	1 (4 %)	1 (4 %)	5 (22 %)	0
WTAP_WTBT_up (293)	43 (15 %)	97 (33 %)	6 (2 %)	25 (9 %)	30 (10 %)	20 (7 %)
WTAP_WTBT_dn (467)	62 (13 %)	186 (40 %)	29 (6 %)	84 (18 %)	70 (15 %)	19 (4 %)
WTAP_WTBT Total (760)	105 (14 %)	283 (37 %)	35 (5 %)	109 (14 %)	100 (13 %)	39 (5 %)
WTBT_WTNT_up (312)	62 (20 %)	92 (30 %)	10 (3 %)	36 (12 %)	31 (10 %)	17 (5 %)
WTBT_WTNT_dn (680)	73 (11 %)	227 (33 %)	15 (2 %)	112 (17 %)	91 (13 %)	37 (5 %)
WTBT_WTNT Total (992)	135 (14 %)	319 (32 %)	25 (3 %)	148 (15 %)	122 (12 %)	54 (5 %)
WTAP_MTAP_up (74)	17 (23 %)	18 (24 %)	2 (3 %)	9 (12 %)	8 (11 %)	0
WTAP_MTAP_dn (144)	7 (5 %)	41 (29 %)	3 (2 %)	9 (6 %)	15 (10 %)	12 (8 %)
WTAP_MTAP Total (218)	24 (11 %)	59 (27 %)	5 (2 %)	18 (8 %)	23 (11 %)	12 (6 %)
WTBT_MTBT_up (192)	29 (15 %)	77 (40 %)	20 (10 %)	34 (18 %)	28 (15 %)	2 (1 %)
WTBT_MTBT_dn (522)	43 (8 %)	194 (37 %)	4 (1 %)	50 (10 %)	63 (12 %)	32 (6 %)
WTBT_MTBT Total (714)	72 (10 %)	271 (38 %)	24 (3 %)	84 (12 %)	91 (13 %)	34 (5 %)
MTAP_MTBT_dn (20)	4 (20 %)	10 (50 %)	3 (15 %)	6 (30 %)	0	2 (10 %)

### Prepenetration appressoria rely on stored nutrients, and experience significant oxidative stress

A Gene Ontology (GO) analysis of transcripts that were differentially expressed during the transition from pre-penetration WT appressoria (AP) to biotrophy (BT) was performed by using Blast2GO [38] (Additional file 2: Table S5). An enhanced expression of genes in AP encoding proteins associated with oxidation and export of potentially toxic compounds and defense against stress, especially oxidative stress, suggests that the fungus is exposed to antimicrobial host defenses even at this early stage. Genes involved in proline metabolism were elevated in AP relative to BT (Additional file 2: Table S5). In *C. trifolii*, proline was a potent antioxidant under nutrient-limiting conditions [39]. Proline accumulation may protect appressoria from ROS accumulation during pre-penetration stages. ROS production by the host cells can be detected during all stages of development in infected leaf tissues [11, 28], but the specific fungal response to oxidative stress appears to be stage-specific, with different antioxidant genes expressed at different phases of development. ROS are also important regulators of normal fungal development, and induction of some antioxidant genes may be related to developmental regulation of the fungus, rather than a response to host defenses [40, 41].

Before host penetration, plant pathogens face starvation conditions and rely on the metabolism of stored compounds. Carbon and nitrogen starvation have been reported as environmental cues for the expression of many pathogenicity-associated genes [42–44]. Spores of *C. graminicola* contain large lipid bodies, which provide the nutrients required for germination, appressorial maturation, and host penetration [45]. Expression of isocytrate lyase (ICL) (GLRG_04226), a key enzyme of the glyoxylate cycle, was significantly increased in AP relative to BT and NT. ICL is elevated during early infection stages in several other pathogenic fungi, and it is essential for pathogenicity of the hemibiotrophs *Magnaporthe oryzae* and *Leptosphaeria maculans* [46–48]. ICL and other components of the glyoxylate cycle are required for appressorial maturation and function in both *C. orbiculare* and *M. oryzae* [49, 50] and play important roles in pathogenicity of many fungal and bacterial pathogens of both plants and animals [51].

Genes involved in transferase activities, including dimethylallyl tryptophan synthases (DMATs) and other aromatic prenyltransferases, were overrepresented in AP relative to BT (Additional file 2: Table S5). Other SM associated genes, including genes involved in melanin biosynthesis, were also more highly expressed. Some of these SM may play roles in stress mitigation during AP, while others may be important for inducing host susceptibility. Several genes associated with adhesion to hydrophobic surfaces (PF12296) were more highly expressed during AP (Additional file 2: Table S5). These proteins may be involved in appressorial maturation and attachment, which are important for appressorial function and signaling [52].

### The biotrophic phase is characterized by an increase in secretory activity, especially the production of secreted proteases

The transition from AP to BT was characterized by an increase in the expression of genes encoding secreted proteins (Additional file 2: Table S5). These included proteins that have been implicated in interactions with other organisms, and numerous secreted proteases belonging to classes that are known to play important roles in plant pathogenicity (Additional file 2: Table S5) [26, 53–55]. Subtilisins are serine proteases that can degrade cell wall proteins and plant defense proteins [56]. Targeted deletion of the subtilisin gene *Spm1* severely compromised pathogenicity of *M. oryzae* to rice plants [57] Another up-regulated protease was a metalloprotease belonging to the *M. oryzae AVR-Pita* avirulence gene family [58]. Carboxypeptidases and aspartic proteases were also represented. Mutant rice plants that transiently expressed a carboxypeptidase inhibitor from potato were highly resistant to infection by the blast fungus *M. oryzae* and the root pathogen *Fusarium verticillioides* [59]. Aspartic proteases have been identified in *Botrytis cinerea*-inoculated carrots, cabbage and grapes [53]. Application of the purified enzymes from *B. cinerea* induced cell death in carrot cell cultures, and inhibition of the enzymatic activity significantly reduced virulence. It appears that in *C. graminicola*, production of a wide variety of secreted proteases is an important feature of biotrophic establishment and colonization. Many of these proteases are likely to target host defense mechanisms, as has been observed in other pathosystems [26].

In both *C. higginsianum* and *C. orbiculare,* biotrophic hyphae are proposed to function primarily as secretory organs for the production of SSP effectors and SM for modification of the plant environment, induction of compatibility, and suppression of programmed host cell death (PCD) [31, 60]. Host cell death could be delayed directly, by targeting host PAMP receptors, and also indirectly, by down-regulating the production of potential elicitors of PCD.

Among the 23 genes that were specifically down-regulated during BT in *C. graminicola*, some were homologs of genes encoding known toxin-associated proteins, eg. *TRI12* from *F. sporotrichoides* [61], *AKT1* from *Alternaria alternata* [62], and *DEP4* from *A. brassicciola* [63] (Table 2). There was also a homolog of a cytochrome P450 monoxygenase gene from *F. graminearum* (FGSG_00007) that, when knocked out, resulted in greatly increased toxin production and virulence of that pathogen to wheat [64]. It is possible that these genes are down-regulated in biotrophic hyphae to avoid triggering host cell death prematurely. Among the other genes that were specifically reduced in expression were two transcription factors, suggesting the possibility of phase-specific negative regulation of transcription.

The transition to BT was associated with an increase in expression of genes encoding secreted hydrolases, including cutinases, pectate lyases, and chitin deacetylases (Additional file 2: Table S5). Cutinases are involved in the interactions of fungi with host cuticle, and play an important role in signaling to trigger fungal development, and in the activation of host defense [65]. Pectate lyases target pectin, the major polysaccharide comprising the middle lamella, and they are important pathogenicity factors in several pathosystems [66–68]. Chitin deacetylases convert chitin in the cell walls of primary hyphae of *Colletotrichum* to chitosan, and it has been suggested that this prevents it from being recognized by the host plant and triggering PAMP-mediated resistance during biotrophic colonization [69, 70].

Expression of multiple acid phosphatases was increased in BT relative to AP. Phytases are one class of acid phosphatases that convert organic forms of phosphorous into inorganic phosphate that can be utilized by the fungus [71]. A predicted extracellular phytase (GLRG_06496) and multiple membrane-bound phosphate transporters (GLRG_01384, GLRG_03681, GLRG_006247, GLRG_09610, and GLRG_10529) were significantly increased in expression during both BT and NT relative to AP. This suggests that the *in planta* environment is relatively poor in phosphorous [72]. A previous study identified phytase as a highly expressed activity in biotrophic hyphae purified from maize stalks by laser capture [73]. The siderophore biosynthesis gene SID1 (GLRG_06540) and the gene encoding NPS6, which is responsible for synthesis of secreted siderophores (GLRG_08065), were both more highly expressed in BT versus AP, rather than being specifically down-regulated as previously reported [12]. Aryl-sulfatase, responsible for sulfur catabolism, was reportedly highly expressed during penetration, and then progressively reduced in expression during biotrophy and necrotrophy in *C. gloeosporioides* [74], but our RNA-Seq data did not support differential expression of the homolog of this gene (GLRG_11452) in *C. graminicola*. The differences in our findings may relate to differences in the strains or species, or in the methods used (RNA-Seq versus semi-quantitative PCR or fluorescent reporters), or to difficulties with precisely and reproducibly identifying the biotrophic phase of development, which is non-synchronous and relatively brief. Our data suggest that biotrophic hyphae of *C. graminicola* actively assimilate phosphorus, sulfur, and iron from the plant host.

Nearly all of the genes that were increased in expression in BT relative to AP were also increased in NT. Only five genes were increased in expression specifically during BT. One of these was associated with vitamin B6 biosynthesis. Genes involved with biosynthesis of other B vitamins, B1 and B12, were significantly increased during both BT and NT relative to AP. The B vitamins are cofactors of many different enzymes, including some involved in carbon and amino acid metabolism [75, 76], suggesting that there is an increase in these activities during the growth of *C. graminicola in planta*. Vitamin B1 biosynthetic genes were significantly induced *in planta* in the biotrophs *Puccinia triticina* and *Uromyces fabae* [77, 78]. Vitamin B6, also known as pyridoxine, has also been associated with antioxidant activities and resistance to oxidative stress in plant and fungi [79, 80]. Active detoxification mechanisms could be important to overcome defense mechanisms and establish a successful biotrophic interaction.

Two other biotrophy-specific genes encode members of large classes of membrane proteins that may play roles in signaling. One was a potential G-protein coupled receptor protein in the Pth11 family [81, 82]. This family is very large in *C. graminicola*, with 50 members. Ten Pth11-family genes were differentially expressed in the WT, with eight transcribed early, and two late during the infection. These CFEM-domain receptors may play roles in the specific recognition of plant signals, and mediation of developmental transitions during the *Colletotrichum*-maize interaction. The second biotrophy-specific signaling gene encoded a putative heterokaryon incompatibility protein. Two additional HET genes were up-regulated during both BT and NT, relative to AP. HET proteins interact in heterologous pairs to trigger cell death in fungi, including *C. graminicola* [83]. Expression of HET proteins during growth *in planta* may serve to regulate intra-mycelial interactions, and protect the colonized tissues from encroachment by other *C. graminicola* strains.

### The transition to necrotrophy was characterized by an increase in production of hydrolytic enzymes, and in the utilization of carbon and nitrogen from the host

The transition to NT in the WT was associated primarily with an increase in activities that have been related to degradation of host cell walls [84] (Additional file 2: Table S6). These included hydrolase activities (including cellulases, laccases, and peptidases), carbohydrate catabolism, and cellulose binding (Additional file 2: Table S6). Cell wall degrading enzymes (CWDE) are utilized for host penetration and colonization, and also to obtain nutrients from plant polymers [84]. Necrotrophic plant pathogens normally secrete large quantities of CWDE during host colonization, whereas biotrophs produce relatively few [17, 21] Categories related to stress response, particularly hypoxia, were decreased in NT compared with BT, signifying that the necrotrophic hyphae were experiencing less oxygen deprivation than the biotrophic hyphae. This may be related to the relative disorganization of the host tissues in NT versus BT, and lack of a host membrane surrounding the necrotrophic hyphae.

There are a large number of *C. graminicola* genes that were primarily or only expressed during NT (late genes) (Additional file 2: Table S3). A majority of these encoded CWDE of various classes. This is consistent with cytological evidence that dissolution of host cell walls becomes evident only after the transition to necrotrophy [7, 11]. Among the late genes were two glutamate importers (GLRG_03954 and GLRG_04076), suggesting increased availability of this preferred nitrogen source after induction of host cell death. Availability of preferred carbon sources is also indicated by a significant decrease in the expression of ICL during NT relative to AP and BT. These results indicate that necrotrophic hyphae of *C. graminicola* are more active than biotrophic hyphae in the uptake and utilization of nutrients from the host tissues. Degradation of the tissue during this phase likely facilitates this uptake by making nutrients more accessible and available to the fungal mycelium.

It is important to note that 1131 (42 %) of the differently expressed genes were not annotated by Blast2GO (Table 5). Among these non-annotated genes, 339 are predicted to encode secreted proteins, and another 208 of the unassigned genes are predicted to encode plasma membrane proteins (Table 5).

**Table 5:** Number of genes in each comparison that were annotated using Blast2GO

	Total genes		Percentage	
Stage transition	Annotated	Not annotated	Annotated	Not annotated
WTAP_WTBT_up	164	129	56	44
WTAP_WTBT_dn	327	140	70	30
WTBT_WTNT_up	151	161	48	52
WTBT_WTNT_dn	439	241	65	35
MTAP_MTBT_dn	15	5	75	5
WTAP_MTAP_up	31	43	42	58
WTAP_MTAP_dn	86	58	60	40
WTBT_MTBT_up	128	64	67	33
WTBT_MTBT_dn	232	290	44	56

### Patterns of expression of predicted effector genes

During host colonization, biotrophic plant pathogens produce SSP effectors that suppress host defense responses and reprogram host metabolism [19, 23, 85–87]. Putative effector proteins are produced by appressoria, and can be detected in the appressorial pore of *C. higginsianum* prior to penetration [31, 88]. It was proposed that these function similarly to SSP effectors in biotrophs, to predispose the living host cells for fungal invasion. Other SSP effectors are involved in the induction of PCD of plant cells in necrotrophic and hemibiotrophic pathogens [31, 89, 90]. A total of 341 differentially expressed genes in *C. graminicola* are predicted to encode SSP (defined here as ≤ 300 amino acids), and 128 are predicted to encode SSP-CR. Most of these genes were not annotated by Blast2Go (Table 6). Distinct subsets of SSP genes were expressed during each phase of development *in planta* in *C. graminicola*. Analysis of the expression of six putative SSP effectors by using qRT-PCR validated their patterns of expression as indicated by the RNA-Seq data (Additional file 1: Figure S3). Expression in appressoria formed *in planta* vs. in vitro confirmed that the expression of most of them was induced *in planta* (Additional file 1: Figure S3).

**Table 6:** Annotation of differentially expressed genes encoding secreted proteins

	Total (Annotated + Non-annotated)					Non-annotated				
	Total	Secreted	SSP	SSP-CR	Membrane	Total	Secreted	SSP	SSP-CR	Membrane
TOTAL GENOME	12,006	1650 13.7 %^a^	687 5.7 %^a^	251 2.1 %^a^	1689 14.1 %^a^	NA	NA	NA	NA	NA
WTAP_WTBT_up	293	84 28.7 %^a^	43 14.7 %^a^	18 6.1 %^a^	70 23.9 %^a^	129 44 %^b^	43 51.2 %^b^	30 69.8 %^b^	13 72.2 %^b^	30 42.9 %^b^
WTAP_WTBT_dn	467	189 40.1 %	62 13.3 %	21 4.5 %	103 22.1 %	140 30 %	55 29.1 %	33 53.2 %	14 66.7 %	28 27.2 %
WTBT_WTNT_up	312	121 38.8 %	62 19.9 %	31 9.9 %	64 20.5 %	161 51.6 %	67 55.4 %	48 77.4 %	25 80.6 %	32 50 %
WTBT_WTNT_dn	680	208 30.6 %	74 10.9 %	23 3.4 %	127 18.7 %	241 35.4 %	53 25.5 %	32 43.2 %	14 60.9 %	36 28.3 %
MTAP_MTBT_dn	20	10 50 %	4 20 %	1 5 %	1 5 %	5 25 %	0	0	0	1 100 %
WTAP_MTAP_up	74	28 37.8 %	17 23 %	9 12.2 %	10 13.5 %	43 58.1 %	16 57.1 %	13 76.5 %	7 77.8 %	5 50 %
WTAP_MTAP_dn	144	27 18.8 %	7 4.9 %	1 0.7 %	17 11.8 %	58 40.3 %	11 40.7 %	6 85.7 %	1 100 %	5 29.4 %
WTBT_MTBT_up	192	98 51 %	29 15.1 %	12 6.3 %	44 22.9 %	64 33.3 %	32 32.7 %	18 62.1 %	9 75 %	7 15.9 %
WTBT_MTBT_dn	522	116 22.2 %	43 8.2 %	12 2.3 %	111 21.3 %	290 55.6 %	62 53.4 %	33 76.7 %	11 91.7 %	64 57.7 %
TOTAL	2704	881 32.6 %	341 12.6 %	128 4.7 %	547 20.2 %	1131 41.8 %	339 38.5 %	213 62.5 %	94 73.4 %	208 38 %

^a^Percentage of total genes within the same comparison or dataset

^b^Percentage of total differentially expressed genes in the same category within the comparison

Effectors that play direct roles in host-pathogen recognition are often lineage-specific, as a result of diversifying selection and gain-loss evolutionary dynamics [91–93]. Eighteen effectors that appear to be unique to *C. graminicola*, aka lineage-specific (LS)-SSPs, were differentially expressed during one or more developmental transitions, and five were among the most highly expressed genes in at least one developmental phase (Table 7; Additional file 2: Table S7). Two thirds of the differentially expressed LS-SSP genes in *C. graminicola* were expressed during earlier phases of development, suggesting that penetration and the establishment of biotrophy are primary points of host recognition during the anthracnose disease interaction.

**Table 7:** Categories of fungal effectors and effector families in the *C. graminicola* genome and transcriptome

	TOTAL GENOME	TOP100 WTAP	TOP100WTBT	TOP100WTNT	TOP100MTAP	TOP100MTBT	WTAP_WTBT_Up	WTAP_WTBT_Dn	WTBT_WTNT_Up	WTBT_WTNT_Dn	MTAP_MTBT_Dn	WTAP_MTAP_Up	WTAP_MTAP_Dn	WTBT_MTBT_Up	WTBT_MTBT_Dn
Total SSP	687	18	11	8	13	14	43	62	62	75	4	17	7	29	44
SSP-CR	251	12	6	3	9	7	18	21	31	23	1	9	1	12	12
Lineage-specific SSP	54	3	1	0	2	4	3	1	9	3	0	5	0	0	1
GH61 motif SSP^a^	14	0	0	1	0	0	0	3	0	8	0	0	1	1	1
CFEM motif SSP^b^	11	0	0	0	0	0	1	0	1	0	0	0	1	0	2

^a^SSP that belong to family 61 of the Glycosyl Hydrolase Cazymes. This is a very large family of fungal enzymes that is thought to play an important role in lignocellulose degradation. These effectors are expressed later during infection, primarily during NT

^b^SSP with a CFEM domain. CFEM proteins contain a conserved domain that includes eight cysteine residues. Many fungal proteins with CFEM domains have been shown to have roles in pathogenicity

Some of the differentially expressed and/or highly expressed putative effectors belong to families that are conserved in other fungi (Table 8). Homologs of BAS2 and BAS3 genes, originally identified as two of the most highly expressed genes during biotrophic colonization of rice plants by *M. oryzae* [94] were also among the most highly expressed genes during AP and BT in *C. graminicola*. Homologs of the *M. oryzae* GAS1 and GAS2 genes, expressed specifically in appressoria of that fungus [95], were also up-regulated in appressoria of *C. graminicola*. The GAS1 and GAS2 proteins are both required for host penetration by *M. oryzae*. Members of a class of conserved effectors containing lysin motifs (LysM) are believed to sequester chitin fragments from pathogenic fungi, thus avoiding detection by the host [27, 96–98]. Two LysM SSP genes are highly expressed during AP and BT in *C. graminicola*. One of these (GLRG_02947) is a homolog of the *M. oryzae* SLP1 gene, which is produced at the interface between the fungal cell wall and the host cell plasma membrane during biotrophic invasion [97]. SLP1 was necessary for virulence of *M. oryzae* to rice. In addition to these, there were many other SSPs that were shared by *M. oryzae* and *C. graminicola*, including a small number that seemed to be specific only to those two genera, suggesting the possibility of functional conservation. Although these two species are only distantly related, they have very similar modes of infection and hemibiotrophic colonization.

**Table 8:** Conserved fungal effectors in the *C. graminicola* transcriptome

	TOP100 WTAP	TOP100WTBT	TOP100WTNT	TOP100MTAP	TOP100MTBT	WTAP_WTBT_Up	WTAP_WTBT_Dn	WTBT_WTNT_Up	WTBT_WTNT_Dn	MTAP_MTBT_Dn	WTAP_MTAP_Up	WTAP_MTAP_Dn	WTBT_MTBT_Up	WTBT_MTBT_Dn
BAS2 (GLRG_06284)	X*			X				X						
BAS3 (GLRG_00201)	X	X		X	X			X						
GAS1 (GLRG_08276)							X							
GAS1 (GLRG_08941)						X								X
GAS1 (GLRG_09110)				X	X			X						
GAS2 (GLRG_03323)						X								X
ClH1 LysM (GLRG_02947)	X	X						X			X			
ClH1 LysM (GLRG_07767)	X					X		X			X			
ChNLP2 (NPP1) (GLRG_11600)							X						X	
NIS1 (GLRG_05338)	X	X	X	X	X									

* An “X” indicates that the gene was included in that group

Hemibiotrophic pathogens produce SSPs during later phases of infection that induce PCD of host cells, and are thought to mediate the switch to necrotrophy [31, 86, 90]. For example, there are six genes in *C. higginsianum* encoding proteins that belong to the *NPP1* family of PCD-inducing effectors found in *Phytophthora* species [26]. There are homologs in *C. graminicola* for all but one of these *C. higginsianum* proteins (Table 8). *C. graminicola* homologs of ChNLP2, ChNLP3 and ChNLP5 are differentially expressed. Only CgNLP1 and CgNLP2 in *C. graminicola* share the amino acids residues crucial for PCD-inducing activity [99, 100]. CgNLP2 is most highly expressed during NT. In contrast, the CgNLP3 and CgNLP5 transcripts are more abundant during AP, like their homologs in *C. higginsianum*. Another conserved SSP gene, GLRG_01192, is a homolog of CgEC91, a hypersensitive-response inducing protein effector, induced during the switch to necrotrophy in *C. higginsianum* [31]. Expression of this gene was increased in *C. graminicola* BT relative to AP.

The patterns of expression of SSPs in *C. graminicola* suggest that, as in other hemibiotrophic pathogens, “early” SSPs, produced by appressoria and biotrophic hyphae, promote compatibility by subverting host recognition and PCD, while “late” SSPs, produced late during biotrophic development, are involved in the switch to necrotrophy by promoting host PCD.

### Patterns of expression of predicted secondary metabolism (SM) clusters

The *C. graminicola* genome is unusually rich in SM genes [15]. SM genes generally occur as part of co-regulated gene clusters. Forty-two predicted SM clusters are encoded by the *C. graminicola* genome [15]. The RNA-Seq data provided evidence for co-regulation of only seven of these clusters *in planta*, defined as having most or all of the genes in the cluster significantly differentially expressed, and with the same pattern of expression (Additional file 2: Table S8).

**Fig. 5:**
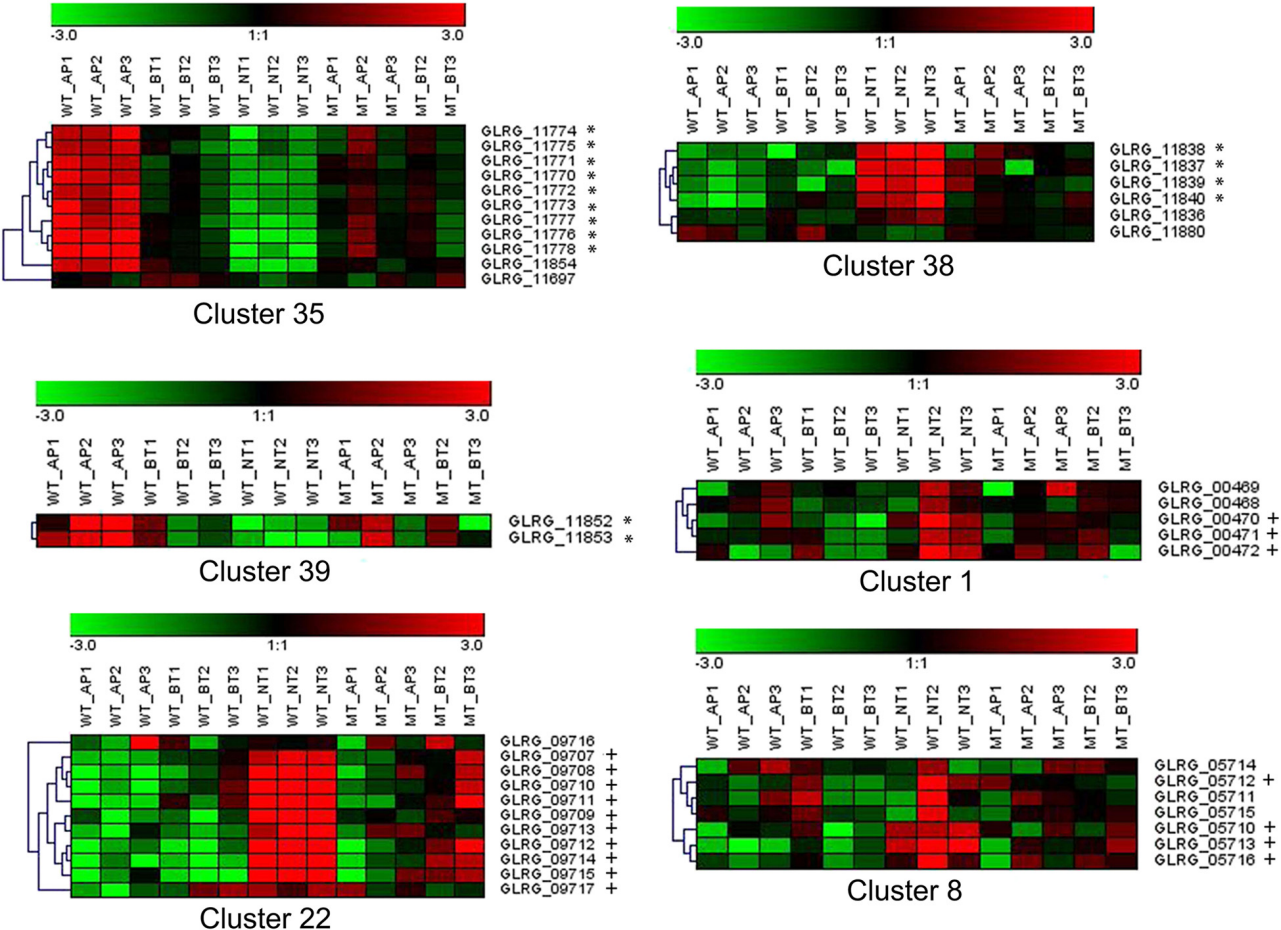
Heatmaps of gene expression of co-regulated SM clusters. Transcript representation is shown as fold changes in each repetition (Log2) relative to the average number of normalized reads for each gene across all stages. * Expression significantly higher in AP (AP-BT_down) ^+^ Expression significantly higher in NT (BT-NT_up)

Distinct subsets of SM-associated genes were expressed across different developmental phases of *C. graminicola in planta*. Clusters 35 (Polyketide Synthase, PKS) and 39 (DMAT) were preferentially expressed in AP, while clusters 1 (Nonribosomal Peptide Synthetase, NRPS), 8 (PKS), 22 (PKS-NRPS hybrid), and 38 (PKS) were more highly expressed during NT (Fig. 5). Expression patterns for six potential SM genes from three of these clusters were confirmed by qRT-PCR analysis (Additional file 1: Figure S4). Most of the differentially expressed SM-associated genes were more highly expressed in appressoria *in planta*, relative to IV-AP, in all three strains (Additional file 1: Figure S4).

Expression of SM genes during NT is expected, given that SM are usually associated with necrotrophic plant pathogens. However, induction of SM during earlier, biotrophic stages of development has been described in other intracellular hemibiotrophs including *C. higginsianum* [15]; *C. orbiculare* [60]; and *M. oryzae* [101, 102] and it has been suggested that SM expressed during early infection stages act to suppress host defenses, rather than to kill tissues. SM expressed later in the infection process may induce host PCD, or protect necrotic tissues from microbial competitors.

The identities of the products generated by most of the differentially regulated *C. graminicola* SM clusters can only be guessed, since the clusters do not closely resemble known clusters from other fungi. However, cluster 38 was an exception because it is identical, both in gene content and in gene order, to the RADS cluster of *Pochonia chlamydosporia* [103], which is responsible for production of the antifungal SM radicicol (aka. monorden). Monorden, and its biosynthetic intermediates monocillins I, II and III, [104], are among the few known fungal metabolites that are produced by *C. graminicola* in maize stalks, and in vitro analyses demonstrated their antifungal activity against other maize stalk-rot and foliar pathogens [105]. Monorden inhibits heat-shock protein (Hsp) 90, by competition with ATP for the binding site required for its activation [106]. It was suggested that *C. graminicola* may secrete these SM products during early penetration and biotrophic stages, in order to suppress basal host defense responses. However, our transcriptional analysis revealed that cluster 38 is significantly induced during WTNT, suggesting instead that monorden plays a role in defending necrotic tissue from microbial competitors.

### Analysis of the MT transcriptome provides additional clues to the nature of biotrophy

Very few biotrophy-specific genes could be identified based on analysis of the WT RNA-Seq data alone. This is probably because BT consists of a mixture of cell types, including numerous pre-penetration appressoria, biotrophic primary hyphae that are just entering living host cells, and intercalary primary hyphae behind the advancing colony front that are occupying cells that are already dead or dying. Nearly all of the genes that were increased in expression in BT relative to AP were also increased in NT. NT is also a mixed culture, with necrotrophic hyphae produced in the center of the colony, but persistence of biotrophy at the colony edges. This lack of synchronicity would be expected to mute potential cell-specific differences in gene expression. Penetration and the establishment of biotrophic hyphae in the living host cell are of great interest as potential determinants of the disease outcome, but these processes are transient and nonsynchronous, and thus the transcriptional activities associated with them are not clearly revealed by the WT RNA-Seq analysis.

The MT strain allowed us to dissect the nature of BT and biotrophic establishment in *C. graminicola* in more detail. The MT is blocked early during the production of the primary hyphae: it does not advance to adjacent cells and thus, MTBT consists only of the initial invasive primary hyphae, and includes no intercalary hyphae [11]. Only 20 genes were differentially expressed in the transition from MTAP to MTBT, compared with 760 in the WT transition. EdgeR MDS plots indicated that MTBT was more similar to WTAP and MTAP than to WTBT. Patterns of the most highly expressed genes in each condition also supported this conclusion (Additional file 2: Table S7).

The establishment of BT is expected to involve the activities of numerous SSP effectors and SM that facilitate invasion by promoting susceptibility and inhibiting host PCD. Among the 20 genes that were upregulated in MTBT relative to MTAP, two were members of the DMAT SM cluster 22 (Table 3). There were also four SSPs. One is a homolog of the glycosyl hydrolase *XYL2* from *C. carbonum*. In *M. oryzae* there was a reduction in virulence when the homolog of this gene was knocked out [107]. GLRG_06286 encodes a secreted metalloprotease and is homologous to *MEP1* from *Coccidioides posadasii*. The MEP1 protein prevents host detection by digesting surface antigens from mice cells. When this gene was mutated, virulence was reduced [108]. The product of this gene in *C. graminicola* may be involved in blocking activity of PAMP receptors of maize. The remaining two differentially expressed SSPs are uncharacterized. One is conserved in multiple *Colletotrichum* species, while the other is found only in *C. graminicola* and its close relative *C. sublineola*. All four SSP genes are also increased in expression in WTBT compared with WTAP, and remained up-regulated in WTNT, suggesting that their function is not specific to penetration or to the establishment of biotrophy. A gene homologous to *ChLae1*, a master regulator thought to function by altering heterochromatin, and that contributes to host selective toxin production, pathogenicity, and adaptation to oxidative stress in *Cochliobolus heterostrophus* [109], was also upregulated in MTBT relative to MTAP. This suggests that the early transition to BT involves chromatin remodeling and a resulting shift toward expression of pathogenicity-specific genes.

Most of these 20 genes were also more highly expressed in WTBT versus WTAP, suggesting that the transition to biotrophy is initiated normally in the MT. There were five exceptions: One of these genes encoded a homolog of *LysF* from *Aspergillus fumigatus*, which functions in lysine biosynthesis and was necessary for pathogenicity of that fungus [110]. Another gene encoded a sedlin-domain protein. Sedlin is a component of the secretory pathway that functions in protein transport from the endoplasmic reticulum to the golgi [111]. Increased activity of the secretory pathway in biotrophic hyphae would be consistent with their putative function as secretory cells for protein effectors [31, 60]. It is possible that all five of these genes are only transiently up-regulated during the establishment of biotrophy, and that this was not detected in the more heterogeneous WTBT samples.

Comparison of WT samples to MT samples collected at the same stage of development provided even more detailed information, specifically about events that occur during the crucial penetration and early, versus late, biotrophic phases.

### Genes expressed during penetration include numerous hydrolases, phytase, and cysteine-rich SSP effectors

Although germination and appressorial induction and maturation occurred at the same rate, penetration by the MT was delayed compared with the WT [11]. Thus, when the MTAP and WTAP samples were collected, the WTAP had already initiated penetration while the MTAP had not. Comparisons between WTAP versus MTAP indicated that WTAP is enriched in genes encoding secreted hydrolases and a phytase, suggesting an increase in these activities coinciding with the initiation of host wall penetration (Additional file 2: Table S9). Seventeen SSPs, nine of which were cysteine-rich, were more highly expressed in the WTAP versus the MTAP (Table 7). Five of these were also among the 100 most highly expressed genes in WTAP (Table 7). Four were early genes, suggesting that they are transiently up-regulated and may have a specific function in penetration, while nearly all of the remainder were expressed at similar levels in both WTAP and WTBT, and then down-regulated during WTNT, suggesting that they have functions that are important both during penetration and biotrophic colonization. These SSPs include the LysM effectors that are presumed to be important for sequestering chitin, and four LS-SSPs that may be involved in specific interactions with the host.

Only seven SSP effectors were more highly expressed in MTAP, suggesting these were induced earlier, prior to the initiation of penetration: none were LS-SSPs, and only one was cysteine-rich (Table 7). Thus, there appears to be a bias toward increased expression of SSPs, especially SSP-CRs and LS-SSPs, coinciding with the initiation of penetration. It is interesting that six of the seven SSP genes that were higher in MTAP versus WTAP were also significantly increased in expression during WTBT and WTNT compared with WTAP. This suggests that these effectors function during all phases of development, not just in appressoria, and also that the expression of these genes might be down-regulated during penetration and early biotrophy.

MTAP was also more active in expression of eight of the genes contained in SM cluster 18. Cluster 18 as predicted contains 24 genes, and ten of these are homologous to genes in cluster 10 in *C. higginsianum* [15]. However, the predicted cluster 18 probably represents two separate clusters in *C. graminicola*, one homologous to the *C. higginsianum* cluster 10 (which our RNA-Seq and qRT-PCR data both confirm is not expressed in any phase); and a second without a homolog in *C. higginsianum*. The eight differentially expressed genes in MTAP belong to the second cluster. The backbone of this second cluster is a non-reducing PKS gene, GLRG_08632. This SM cluster is apparently expressed during appressorial formation prior to the initation of penetration, and produces an unknown SM product.

### Early versus late biotrophy: late biotrophy is characterized by increased expression of genes encoding secreted proteases and transmembrane receptors and transporters

Genes that were more highly expressed in the MTBT versus WTBT were also more likely to be over-expressed in WTAP versus WTBT (Table 9). Furthermore, many of these genes corresponded with “early” appressorium-specific genes in the WT. As previously described, the MT strain fails to establish a successful biotrophic interaction. ICL and malate synthase (GLRG_02557) the key enzymes of the glyoxylate cycle, are more highly expressed in the MT during BT, suggesting that the MT is not efficiently obtaining sugars from the host and still relies on stored compounds. All of this is consistent with the observation that MTBT is blocked in an earlier phase of development than WTBT. Genes that were more highly expressed in WTBT versus MTBT were also more likely to be over-expressed in WTBT versus WTAP, thus these probably represent “late” biotrophy genes, perhaps produced in the intercalary hyphae that are occupying dead and dying host cells behind the advancing colony front.

**Table 9:** Relationships among gene expression patterns for genes that are differentially expressed in both the WTAP vs. WTBT comparisons, and in the WTBT vs. MTBT “Up” or “Down” refers in each case to the first term, in the comparison relative to the second. For example, WTAP_WTBT_up means that the genes are more highly expressed in WTAP than in WTBT

	WTAP_WTBT_up	WTAP_WTBT_dn
WTBT_MTBT_up	0	134
WTBT_MTBT_dn	172	1

In a comparison of GO-Terms associated with WTBT versus MTBT, the WTBT was enriched in extracellular and integral membrane proteins, as well as terms associated with host-pathogen interactions (including several subtilases and proteins involved in peroxide detoxification), transmembrane transport, cellular homeostasis, peptidase activities, and SM (Additional file 2: Table S10). Secreted proteases were over-represented among this “late BT” group, suggesting that these protease functions are more important later in biotrophy versus during the initial invasion of living host cells.

MTBT over-expressed genes involved in the glyoxylate cycle and melanin biosynthesis, an indication that the MTBT sample contained a larger proportion of transcripts from appressoria. This is consistent with the extremely limited development of intracellular hyphae in MTBT compared with WTBT. MTBT over-expressed 43 SSP genes, including 12 SSP-CRs. Twenty-four of the genes are “early” genes that are specifically more highly expressed in WTAP. These are likely to be expressed primarily in the appressoria in the MTBT sample. The other 19 SSP genes were expressed at similar levels in WTAP and WTBT, with five of these decreasing during WTNT, suggesting that they were specific to late AP and early BT.

In comparison with the MTBT, the WTBT was enriched in proteins that might function in communication between host and pathogen, including secreted and integral membrane proteins and proteases potentially involved in evasion of host immune response. Proteins involved in peroxide detoxification that may protect the intracellular hyphae from host ROS, transmembrane transporters to allow uptake and egress of compounds including nutrients or SM, and proteins involved in maintenance of cellular homeostasis, which may preserve hyphal integrity in the dynamic environment of the living host cell, were also over-represented. Twenty-nine SSPs were overexpressed in WTBT including two pectate-lyase domain SSPs that might target the host middle lamella, and the NPP1 homolog ChNLP2, which may be involved in inducing PCD. Cells die rapidly once they have been invaded by *C. graminicola* primary hyphae, possibly due to activation of these necrosis-inducing proteins and production of endogenous elicitors by disruption of the host cellular structure.

### Maize leaf sheaths respond differently to WT and MT infections

It is possible that the delay in penetration by MT appressoria is related to a failure to secrete proteins that are necessary to prepare the epidermal cell for invasion and establishment of biotrophy, and/or to process signals from the host that trigger further development of the pathogen. Host responses to the MT and WT strains at different stages of infection were studied by using qRT-PCR to evaluate expression of maize genes previously associated in the literature with the response to *C. graminicola* or biotrophic pathogens [6, 28, 112, 113].

**Fig. 6:**
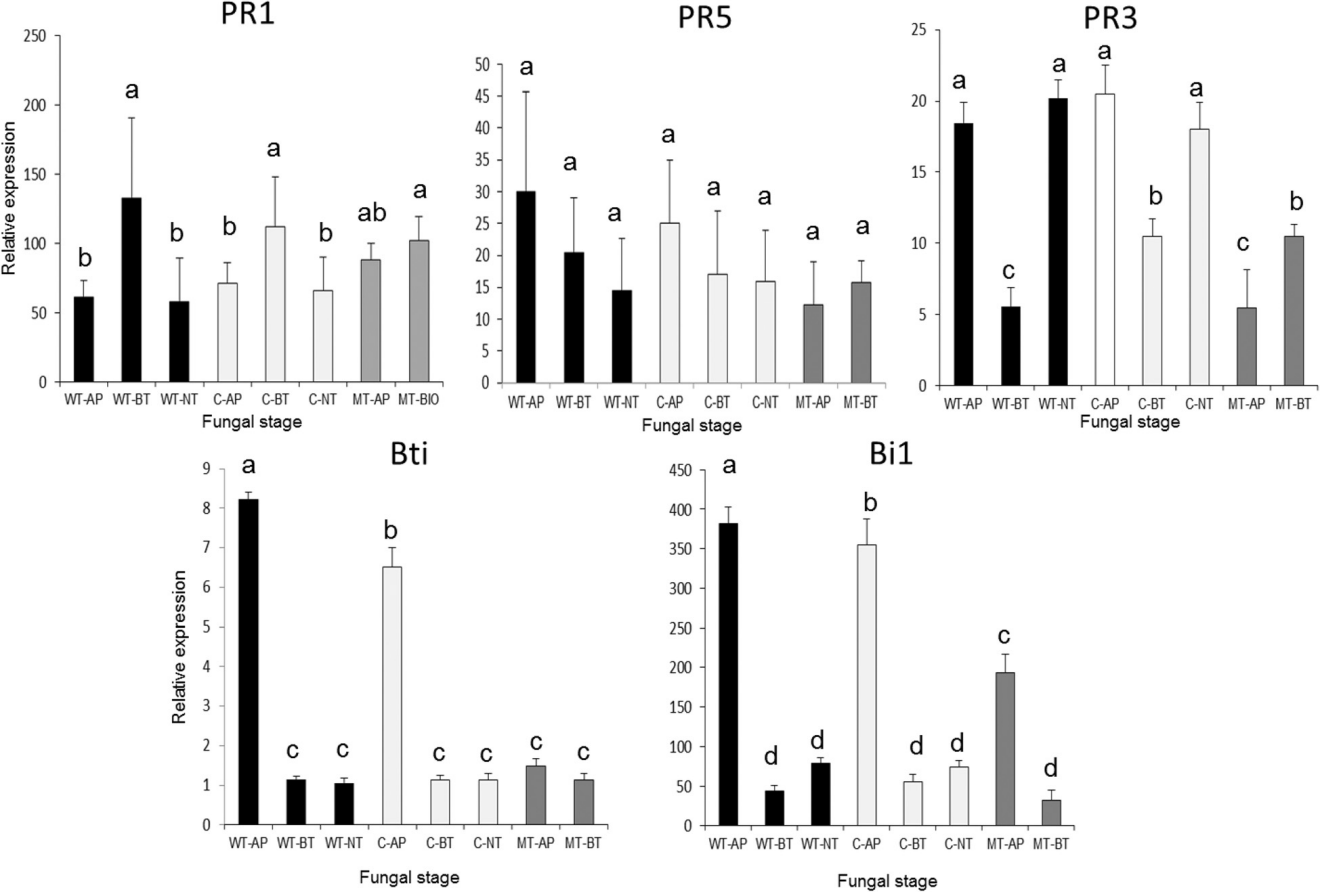
Maize defense responses are activated early into the infection process. Bars represent expression of each gene, relative to mock-inoculated plants. PR: pathogenicity-related. Bti: Bowman-birk trypsin inhibitor (JA marker). Bi1: Bax inhibitor (cell death inhibitor)

Host tissues exposed to MT appressoria generally accumulated fewer disease-associated transcripts (Fig. 6). Plant defense-associated genes are activated in response to fungal elicitors, and differences in expression of these genes could result from the mutant failing to elicit a strong response due to reduced production of elicitors. It was observed previously that ROS production in response to MTAP was also reduced in comparison with WTAP [11].

In barley, expression of the Bax-family PCD inhibitor *Bi1* [114] was induced during infection by the obligate biotrophic pathogen *Blumeria graminis* f.sp. *hordei* [115]. Silencing of *Bi1* significantly reduced infection, while overexpression increased the susceptibility of barley to biotrophs [112, 115]. The maize homolog was increased in expression during WTAP, suggesting that appressoria may produce factors that induce this gene, thereby inhibiting hypersensitive cell death prior to penetration, and preparing the host cell for biotrophic invasion. The maize pathogenicity-related proteins *PR1*, *PR3*, and *PR5* are induced as a result of activation of the salicylic acid (SA) defense pathway [116]. The Bowman-birk trypsin inhibitor (*Bti*) is a marker for induction of jasmonic acid (JA) [117]. SA-dependent pathways are typically deployed against biotrophic plant pathogens, while JA-mediated pathways are usually associated with plant responses against necrotrophic pathogens [118, 119]. The role of SA and JA signaling defense pathways is less clear for hemibiotrophic plant pathogens. In maize seedlings inoculated with *C. graminicola*, SA accumulated at 36 hpi (during biotrophy), while JA could be detected by 96 hpi (during necrotrophy) [6]. Interestingly, expression of *Bti* was significantly induced in maize leaves as early as 24 hpi with the biotrophic *U. maydis*, although silencing of this gene did not affect pathogen colonization [113]. Our data suggest that both defense pathways are activated in leaf sheaths in response to WT appressoria prior to penetration.

## Conclusions

Our study indicates that there is a continuum of activities that occur during colonization of maize by *C. graminicola*, and that the boundaries we have drawn between “AP”, “BT” and “NT” are artificial. About 20 % of the fungal genes are differentially transcribed in “waves” across the processes of appressorial maturation, penetration, and colonization. Expression of genes involved in nutrient uptake and utilization suggest that there is a shift during development from the utilization of stored lipids during appressorial development and early biotrophy toward the uptake and utilization of nutrients including amino acids and sugars from the host during late biotrophy and necrotrophy. Secreted protein, SM protein, and membrane receptor genes are over-represented among the differentially expressed genes, suggesting that the fungus engages in an intimate and dynamic conversation with the host, beginning prior to penetration. This communication process is likely to involve reception of plant signals that trigger subsequent developmental progress in the fungus, as well as the production of signals that induce responses in the host. Later phases of biotrophy are more similar to necrotrophy, with increased production of secreted proteases, inducers of PCD, hydrolases, and membrane bound transporters for the uptake and egress of potential toxins, signals, and nutrients. The initiation of biotrophy on the other hand, when the fungus establishes itself in living host cells, is similar to the penetration phase that occurs late during appressorial development, with increased production of hydrolases targeting the host cell surface and involved in signaling and modification of fungal cell wall to avoid triggering host recognition, as well as the secretion of many SSPs, particularly lineage specific and cysteine-rich SSPs, that are likely to be involved in suppression of host defense responses, including hypersensitive cell death. The mutant provides us with a “snapshot” of the transcriptional events that occur during the critical points of penetration and biotrophic establishment, as it appears to pause the infection process at this phase so that it can be captured by the transcriptome analysis. Identification of a series of highly expressed genes that can serve as markers for different phases of disease development is possible based on the data presented in this study. For example, the following series of effector genes is predicted to represent a temporal sequence that spans the entire infection process: GLRG_08629; GLRG_01735; GLRG_02947; GLRG_00338; GLRG_08975; GLRG_11600; and GLRG_09214. These genes could potentially be used as ‘landmarks’ to identify and compare developmental progress across different host-pathogen strain combinations or different conditions.

These RNA-Seq data do not provide many insights into the root cause of the mutant phenotype, since most genes appear to be transcribed normally in the MT, including the mutant *Cpr1* gene itself. The mutant phenotype is probably related to post-transcriptional events, which must await further study. Nonetheless, detailed investigation of the genes that are differentially expressed between the MT and WT can help to identify potential virulence factors (e.g. SM and SSPs), receptors, and transcriptional regulators that play critical roles in the establishment of biotrophy, and can also help to differentiate genes that are expressed during invasion of living host cells from genes that are expressed later in biotrophy, in cells that are dead and dying, in preparation for the switch to necrotrophy. These genes must be subjected to further study to elucidate their precise roles, and to determine if they represent viable targets for disease management.

## Methods

### Plants and fungal strains

The susceptible maize inbred Mo940 was used for this study. Plants were grown to the V3 stage in the greenhouse, with a 14-h day length, in 3.8 × 21 cm plastic Conetainers (Super SC-10 UV stabilized Stuewe & Sons, Inc. Oregon, USA), containing a mixture of three parts Pro-Mix BX (Premiere Horticulture, Ltd, Riviere du Loup, PQ, Canada) to two parts of sterile topsoil. Plants were watered daily to saturation and fertilized two to three times per week with a solution of 150 ppm of Peters 20-10-20 (Scotts-Sierra Horticultural Products Co., Marysville, OH), beginning one week after germination.

*C. graminicola* strain M1.001 was the wild type (WT) [15]. The mutant (MT) was derived from M1.001 by restriction-enzyme mediated insertional mutagenesis [36, 37]. The MT is nonpathogenic to maize stalks and leaves due to an insertion in the 3′UTR of the *Cpr1* gene, which is predicted to encode one component of the microsomal signal peptidase [36]. A complemented strain (*Cpr1*-C), generated by transformation of the mutant with a 3.6 kb fragment of genomic DNA containing the WT *Cpr1* gene, is fully restored in pathogenicity and comparable to the WT strain both in vitro and *in planta* [36]. The fungal strains were routinely cultured on potato dextrose agar (PDA, Difco) at 23 °C under continuous light.

### Transcriptome profiling

#### Sample preparation and RNA extraction

*C. graminicola* falcate spores were harvested and inoculated on detached maize leaf sheaths as described [11], except that two 20-μl inoculum drops were applied to each leaf sheath, approximately 1 cm from either end, and at least 3 cm apart. Sheaths containing mature pre-penetration appressoria (WTAP, approximately 20 hpi); intracellular biotrophic hyphae, before symptoms or secondary hyphae were visible (WTBT, approximately 36 hpi); or necrotrophic hyphae, in which initial browning of the tissue and secondary hyphae were visible (WTNT, approximately 60 hpi), were collected. The MT does not progress to the production of necrotrophic hyphae, and so only the AP and BT stages were collected for that strain. Thus, our dataset consisted of five genotype-timepoint combinations (i.e. treatments): WTAP, WTBT, WTNT, MTAP, and MTBT. Each infected leaf sheath was inspected under the microscope to confirm the developmental stage. For the BT and NT samples, each sheath was wiped gently with a moistened sterile cotton swab to remove unattached spores and superficial mycelia. The process of trimming, cleaning, and examination did not take more than two minutes per sheath. Six trimmed leaf sheaths (~100 mg) were pooled into each tube, flash-frozen in liquid nitrogen, and maintained at −80 °C until RNA extraction.

Total RNA was extracted by crushing the frozen tissue, followed by grinding in TRIzol (1 ml per 100 mg sample) (Invitrogen). Samples were incubated for 7 h in isopropanol followed by 2 h in 100 % ethanol, both incubated at −20 °C. Samples were purified and treated with DNAse using the RNeasy Plant Mini Kit (Qiagen), according to the manufacturer’s protocol as modified [120], and then resuspended in 50 μl of RNAse-free water. Extracts from approximately 30 pieces of leaf sheath (500 mg) were pooled for each biological replicate. Each biological replicate represented an independent inoculation experiment.

#### RNA sequencing

Three-hundred micrograms of total RNA from three biological replicates of each treatment (with the exception of MTBT, which had only two usable biological replicates) were submitted for sequencing to AgriLife Genomics and Bioinformatics Services (Texas A&M University). Libraries were prepared for each sample by using the Illumina TruSeqTM RNA Sample Preparation Kit and the manufacturer’s instructions. Data were generated from ten lanes of Illumina GAII sequencing, in two separate runs, with barcoding to multiplex biological replicates. Read lengths were 76 bp (including 7 bp for the barcode adaptor). For the first run, eight lanes of a flowcell were used, and lane five was spiked with 1 % PhiX as a control. For the second run, in which additional data for the WTAP was obtained, two lanes were used. A total of four lanes (i.e. technical replicates) of data were generated for WTAP; one lane each was produced for WTBT and WTNT; and two lanes each were produced for MTAP and MTBT. The technical replicates for each treatment were pooled. Data were processed using the Illumina software CASAVA-1.7.0 for base calling and de-multiplexing, and the final results were stored as individual files for each sample in FASTQ format.

#### Alignment to the reference genome

The sequencing reads were mapped to the 12,006 annotated genes of *C. graminicola* strain M1.001 (ACOD01000001) using the CLC Genomics Workbench (GWB) RNA-Seq analysis tool (http://www.clcbio.com). CLC GWB reports gene expression as “Reads Per Kilobase of Transcript per Million Mapped Reads” (RPKM) [121]. The RPKM value has been shown to underperform compared to alternative normalizations that may be applied to “Total Gene Reads” [122]. Pairwise comparison capabilities provided by CLC did not take full advantage of our experimental design. Therefore, we extracted the “Total Gene Reads” field for each replication of each treatment, and subjected this output to further statistical analysis as described below.

#### Modeling

We used a “Mixed Effects” Generalized Linear Model (GLM) to account for biological replication, developmental stage, and genotype effects. The mixed-effect GLM allowed us to leverage data from all developmental stages simultaneously in a unified analysis framework. The GLM improves the statistical power and inference capabilities, and can protect against multiple testing as compared to the pairwise testing paradigm through a single prerequisite “no effects” test. We used the GLM implemented in the R package, edgeR (version 3.0.8) [32]. The methods implemented in edgeR assume that the underlying distribution for the discrete count measures of the next generation sequencing (NGS) is a negative binomial (NB). In conjunction with an internal, model-based normalization method that has been shown to be superior to RPKM, edgeR estimates mean and variance of the NB distribution for each gene [122], and then proceed in a gene by gene manner for experimental design differential expression analysis.

Our GLM specification included main effects and interactions across genotypes (WT and MT) and developmental stage (AP, BT, and NT). Based on these coefficient estimates, six biologically informative contrasts were estimated: (1) WTAP vs. WTBT; (2) WTAP vs. WTNT; (3) WTBT vs. WTNT; (4) WTAP vs. MTAP; (5) WTBT vs. MTBT; (6) MTAP vs. MTBT.

#### Normalization and statistical testing

The filtered genes and their counts were converted into a DGEList R object. The default normalization method of edgeR – Trimmed Mean of M values (TMM) – was then applied to the data using the “calcNormFactors” EdgeR function. TMM uses one sample as the reference, and assumes that most genes are not differentially expressed. As the result of the normalization, the scaling factors are calculated for each sample library [123], and these scale factors are then used for model-based normalization by EdgeR.

The edgeR assumption for the underlying distribution of the RNA-Seq data is a negative binomial distribution. The dispersion for each gene is using the quantile-adjusted conditional maximum likelihood (qCML) implemented by the “estimateGLMTagwiseDisp” EdgeR function. Statistical testing was carried out with the “glmFit” and “glmLRT” R functions. The genes were classified as up- or down-regulated using the “decideTestsDGE” EdgeR function. Multiple testing was addressed using the false discovery rate (FDR) [124] as implemented in the p.adjust R function.

### Heatmaps

Heatmaps were built by using Genesis (Release 1.7.6) [125], and represent log2-fold changes of a transcript in each of the three fungal developmental stages, relative to the average expression across all stages.

### Quantitative RT-PCR

#### RNA extraction from inoculated leaf sheaths

Leaf sheaths were inoculated with WT, MT and complemented mutant strains, incubated, trimmed, and flash-frozen for RNA extraction as described above. Additional treatments were also tested in the (q)RT-PCR experiments. Tissue inoculated with the *Cpr1*-C strain, and sheaths that were mock-inoculated with water, were also collected, trimmed, and frozen for RNA extraction as described previously.

#### RNA extraction from in vitro appressoria

Appressoria of the WT, MT, and *Cpr1-*C strains were produced in vitro on polystyrene Petri dishes as described in [88], with some modifications. *C. graminicola* spores were collected and washed three times, and 40 ml of a spore suspension at a concentration of 2 × 10^4^ spores/ml was added to each Petri dish. Twenty hours later, each plate was inspected under the microscope to verify the presence of mature melanized appressoria. Appressoria were broken and scraped from the bottoms of ten Petri plates, using a sterile culture spreader, into a total of five ml of TRIzol. Appressoria collected from 40 Petri plates were combined into each replicate. RNA purification was performed as described above.

#### cDNA synthesis and cycling reactions

The SuperScript II reverse transcriptase kit (Invitrogen) was used to synthesize the first strand of cDNA from 1 μg of DNase and RNase-treated total RNA in a volume of 20 μl. Primers were designed to amplify 100–200 bp fragments, using PrimerQuest (Integrated DNA Technologies) software. The reaction mix for real-time PCR contained 0.4 mM of each primer, 10 μl of SYBR green PCR Master Mix (AppliedBiosystems), 5 μl of a 1:5 dilution of the cDNA product, and DEPC water to a final volume of 20 μl. Cycling conditions were as follows: 95 °C for 10 min, followed by 40 cycles of 95 °C for 15 s and 60 °C for 1 min. The reactions were carried out in fast 96-well reaction plates on the ABI 7900HT fast RT-PCR system (Applied Biosystems). Fungal transcript levels were normalized by using the fungal actin gene as an internal standard, and relative expression was calculated using the Pfaffl method [126]. Maize genes were normalized against the maize actin gene, and expression was calculated relative to mock-inoculated plants.

### Functional annotation and gene ontology

Nucleotide sequences similar to differentially expressed genes (P < 0.05), were identified by BLASTx searches of the non-redundant database (E value = 1 × 10^−3^) of the Blast2GO suite [38]. Functional characterization and gene ontology (GO) categories for cellular functions, cellular components, and biological processes, were assigned using the Blast2Go platform. The GOSSIP function was utilized to determine GO term enrichment in different comparisons [127]. Manual annotation of specific genes was performed by using BLAST searches against the NCBI databases (E value = 1 × 10^−5^) and InterproScan analysis. Comparisons to *C. higginsianum* were performed by using the tools available on the Broad Institute *Colletotrichum* database (https://www.broadinstitute.org/scientific-community/science/projects/fungal-genome-initiative/colletotrichumgenome-project). Cellular localization of significantly expressed transcripts was predicted by using WoLF PSORT (http://wolfpsort.org/) [128]. To identify protein families, the Pfam database (http://pfam.sanger.ac.uk/) (E value = 1 × 10^−5^) was used [129]. Transporters were predicted using the Transporters Classification Database (http://www.tcdb.org) (E value = 1 × 10^−5^) [130]. The identification of carbohydrate active enzymes (CAZymes) was done using the web resource dbCAN (http://csbl.bmb.uga.edu/dbCAN/annotate.php), an automated CAZyme annotation that is based on the classification scheme of CAZyDB [131, 132]. For the classification of putative secreted proteases, the sequences for the secreted proteins predicted by WoLF PSORT were submitted to MEROPS Batch Blast analysis (http://merops.sanger.ac.uk) [133] as described in O’Connell et al.[15]. BLAST with an e-value cutoff of 1e^−5^ was used to identify candidate *Colletotrichum* pathogenicity proteins with similarity to proteins present in the Pathogen-Host Interaction [5] .

## Additional files

Additional file 1: Figure S1. Supplemental Figures. (DOCX 13389 kb) Additional file 2: Supplemental Data Tables. (XLSX 1099 kb)

## References

[1] Bergstrom GC, Nicholson RL (1999). The Biology of Corn Anthracnose: Knowledge to Exploit for Improved Management. Plant Dis.

[2] Frey TJ (2011). Fitness Evaluation of, a Locus that Confers Resistance to (Ces.) GW Wils. Using Near-Isogenic Maize Hybrids. Crop Sci.

[3] Horbach R (2011). When and how to kill a plant cell: infection strategies of plant pathogenic fungi. J Plant Physiol.

[4] Bancal M-O, Hansart A, Sache I, Bancal P (2012). Modelling fungal sink competitiveness with grains for assimilates in wheat infected by a biotrophic pathogen. Annals of Botany..

[5] Baldwin TK (2006). The Pathogen-Host Interactions Database (PHI-base) Provides Insights into Generic and Novel Themes of Pathogenicity. Mol Plant Microbe Interact.

[6] Balmer D (2013). Induced resistance in maize is based on organ-specific defence responses. Plant J.

[7] Mims CW, Vaillancourt LJ (2002). Ultrastructural characterization of infection and colonization of maize leaves by Colletotrichum graminicola, and by a C. graminicola pathogenicity mutant. Phytopathology.

[8] Politis D, Wheeler H (1973). Ultrastructural study of penetration of maize leaves by Colletotrichum graminicola. Physiol Plant Pathol.

[9] Venard C, Vaillancourt L (2007). Colonization of fiber cells by Colletotrichum graminicola in wounded maize stalks. Phytopathology.

[10] Venard C, Vaillancourt L (2007). Penetration and colonization of unwounded maize tissues by the maize anthracnose pathogen Colletotrichum graminicola and the related nonpathogen C. sublineolum. Mycologia.

[11] Torres MF, Cuadros DF, Vaillancourt LJ (2014). Evidence for a diffusible factor that induces susceptibility in the Colletotrichum–maize disease interaction. Mol Plant Pathol.

[12] Albarouki E (2014). Biotrophy-specific downregulation of siderophore biosynthesis in Colletotrichum graminicola is required for modulation of immune responses of maize. Mol Microbiol.

[13] Crouch J, et al. The genomics of Colletotrichum, in Genomics of Plant-Associated Fungi: Monocot Pathogens2014. Heidelberg, New York, Dordrecht, London: Springer. p. 69–102.

[14] Münch S (2008). The hemibiotrophic lifestyle of Colletotrichum species. J Plant Physiol.

[15] O’Connell RJ (2012). Lifestyle transitions in plant pathogenic Colletotrichum fungi deciphered by genome and transcriptome analyses. Nat Genet.

[16] Markham JE, Hille J (2001). Host‐selective toxins as agents of cell death in plant–fungus interactions. Mol Plant Pathol.

[17] Amselem J (2011). Genomic analysis of the necrotrophic fungal pathogens Sclerotinia sclerotiorum and Botrytis cinerea. PLoS Genet.

[18] Djamei A (2011). Metabolic priming by a secreted fungal effector. Nature.

[19] Doehlemann G (2009). Pep1, a secreted effector protein of Ustilago maydis, is required for successful invasion of plant cells.

[20] Rafiqi M (2012). Challenges and progress towards understanding the role of effectors in plant–fungal interactions. Curr Opin Plant Biol.

[21] Spanu PD (2010). Genome expansion and gene loss in powdery mildew fungi reveal tradeoffs in extreme parasitism. Science.

[22] de Wit PJGM (2009). Fungal effector proteins: past, present and future. Mol Plant Pathol.

[23] Kämper J (2006). Insights from the genome of the biotrophic fungal plant pathogen Ustilago maydis. Nature.

[24] Lo Presti L (2015). Fungal effectors and plant susceptibility. Annu Rev Plant Biol.

[25] Rooney HCE (2005). Cladosporium Avr2 inhibits tomato Rcr3 protease required for Cf-2-dependent disease resistance. Science.

[26] Kamoun S (2006). A catalogue of the effector secretome of plant pathogenic oomycetes. Annu Rev Phytopathol.

[27] de Jonge R (2010). Conserved fungal LysM effector Ecp6 prevents chitin-triggered immunity in plants. Science.

[28] Vargas WA (2012). Plant defense mechanisms are activated during biotrophic and necrotrophic development of Colletotricum graminicola in maize. Plant Physiol.

[29] Wharton PS, Julian AM (1996). A cytological study of compatible and incompatible interactions between Sorghum bicolor and Colletotrichum sublineolum. New Phytol.

[30] Wharton PS, Julian AM, O’Connell RJ (2001). Ultrastructure of the infection of Sorghum bicolor by Colletotrichum sublineolum. Phytopathology.

[31] Kleemann J (2012). Sequential delivery of host-induced virulence effectors by appressoria and intracellular hyphae of the phytopathogen Colletotrichum higginsianum. PLoS Pathog.

[32] Robinson MD, McCarthy DJ, Smyth GK (2010). edgeR: a Bioconductor package for differential expression analysis of digital gene expression data. Bioinformatics.

[33] Gentleman RC (2004). Bioconductor: open software development for computational biology and bioinformatics. Genome Biol.

[34] Team, RC. R Language Definition, 2000, Available from CRAN sites.

[35] Law CW (2014). Voom: precision weights unlock linear model analysis tools for RNA-seq read counts. Genome Biol.

[36] Thon MR (2002). CPR1: a gene encoding a putative signal peptidase that functions in pathogenicity of Colletotrichum graminicola to maize. Mol Plant Microbe Interact.

[37] Thon MR, Nuckles EM, Vaillancourt LJ (2000). Restriction enzyme-mediated integration used to produce pathogenicity mutants of Colletotrichum graminicola. Mol Plant Microbe Interact.

[38] Conesa, A. and S. Götz, Blast2GO: A comprehensive suite for functional analysis in plant genomics. International journal of plant genomics. 2008;2008:12. doi:10.1155/2008/619832. 10.1155/2008/619832 PMC2375974 18483572

[39] Chen C, Dickman MB (2005). Proline suppresses apoptosis in the fungal pathogen Colletotrichum trifolii. Proc Natl Acad Sci U S A.

[40] Egan MJ (2007). Generation of reactive oxygen species by fungal NADPH oxidases is required for rice blast disease. Proc Natl Acad Sci.

[41] Fang GC, Hanau RM, Vaillancourt LJ (2002). The SOD2 gene, encoding a manganese-type superoxide dismutase, is up-regulated during conidiogenesis in the plant-pathogenic fungus Colletotrichum graminicola. Fungal Genet Biol.

[42] Marzluf GA (1997). Genetic regulation of nitrogen metabolism in the fungi. Microbiol Mol Biol Rev.

[43] Coleman M (1997). Starvation-induced genes of the tomato pathogen Cladosporium fulvum are also induced during growth in planta. Mol Plant Microbe Interact.

[44] Pellier AL (2003). CLNR1, the AREA/NIT2‐like global nitrogen regulator of the plant fungal pathogen Colletotrichum lindemuthianum is required for the infection cycle. Mol Microbiol.

[45] Schadeck RJG, Leite B, de Freitas Buchi D (1998). Lipid mobilization and acid phosphatase activity in lytic compartments during conidium dormancy and appressorium formation of Colletotrichum graminicola. Cell Struct Funct.

[46] Idnurm A, Howlett BJ (2002). Isocitrate lyase is essential for pathogenicity of the fungus Leptosphaeria maculans to canola (Brassica napus). Eukaryot Cell.

[47] Lee H-S (2007). Inhibition of the pathogenicity of Magnaporthe grisea by bromophenols, isocitrate lyase inhibitors, from the red alga Odonthalia corymbifera. J Agric Food Chem.

[48] Wang ZY (2003). The glyoxylate cycle is required for temporal regulation of virulence by the plant pathogenic fungus Magnaporthe grisea. Mol Microbiol.

[49] Asakura M, Okuno T, Takano Y (2006). Multiple Contributions of Peroxisomal Metabolic Function to Fungal Pathogenicity in Colletotrichum lagenarium. Appl Environ Microbiol.

[50] Wang Z-Y (2007). Functional analysis of lipid metabolism in Magnaporthe grisea reveals a requirement for peroxisomal fatty acid β-oxidation during appressorium-mediated plant infection. Mol Plant Microbe Interact.

[51] Dunn MF, Ramirez-Trujillo JA, Hernández-Lucas I (2009). Major roles of isocitrate lyase and malate synthase in bacterial and fungal pathogenesis. Microbiology.

[52] Hamer JE, Talbot NJ (1998). Infection-related development in the rice blast fungus Magnaporthe grisea. Curr Opin Microbiol.

[53] Movahedi S, Heale JB (1990). The roles of aspartic proteinase and endo-pectin lyase enzymes in the primary stages of infection and pathogenesis of various host tissues by different isolates of Botrytis cinerea Pers ex. Pers Physiol Mol Plant Pathology.

[54] ten Have A (2004). An aspartic proteinase gene family in the filamentous fungus Botrytis cinerea contains members with novel features. Microbiology.

[55] Valueva TA, Mosolov VV (2004). Role of inhibitors of proteolytic enzymes in plant defense against phytopathogenic microorganisms. Biochemistry (Moscow).

[56] Olivieri F (2002). Characterization of an extracellular serine protease of Fusarium eumartii and its action on pathogenesis related proteins. Eur J Plant Pathol.

[57] Oh Y (2008). Transcriptome analysis reveals new insight into appressorium formation and function in the rice blast fungus Magnaporthe oryzae. Genome Biol.

[58] Khang CH (2008). Genome organization and evolution of the AVR-Pita avirulence gene family in the Magnaporthe grisea species complex. Mol Plant Microbe Interact.

[59] Quilis J (2007). A potato carboxypeptidase inhibitor gene provides pathogen resistance in transgenic rice. Plant Biotechnol J.

[60] Gan P (2013). Comparative genomic and transcriptomic analyses reveal the hemibiotrophic stage shift of Colletotrichum fungi. New Phytol.

[61] Alexander NJ, McCormick SP, Hohn TM (1999). TRI12, a trichothecene efflux pump from Fusarium sporotrichioides: gene isolation and expression in yeast. Mol Gen Genet.

[62] Tanaka A (1999). Insertional mutagenesis and cloning of the genes required for biosynthesis of the host-specific AK-toxin in the Japanese pear pathotype of Alternaria alternata. Mol Plant Microbe Interact.

[63] Wight WD (2009). Biosynthesis and role in virulence of the histone deacetylase inhibitor depudecin from Alternaria brassicicola. Mol Plant Microbe Interact.

[64] Gardiner DM, Kazan K, Manners JM (2009). Novel genes of Fusarium graminearum that negatively regulate deoxynivalenol production and virulence. Mol Plant Microbe Interact.

[65] Serrano, M, et al. The cuticle and plant defense to pathogens. Frontiers in plant science, 2014;5. 10.3389/fpls.2014.00274 PMC4056637 24982666

[66] Ben-Daniel B-H, Bar-Zvi D, Tsror L (2012). Pectate lyase affects pathogenicity in natural isolates of Colletotrichum coccodes and in pelA gene-disrupted and gene-overexpressing mutant lines. Mol Plant Pathol.

[67] Cho Y, et al. A Pectate Lyase-Coding Gene Abundantly Expressed during Early Stages of Infection Is Required for Full Virulence in Alternaria brassicicola. PloS One, 2015. 10(5). 10.1371/journal.pone.0127140 PMC4440746 25996954

[68] Cnossen-Fassoni A (2013). The pectate lyase encoded by the pecCl1 gene is an important determinant for the aggressiveness of Colletotrichum lindemuthianum. J Microbiol.

[69] El Gueddari NE (2002). Developmentally regulated conversion of surface‐exposed chitin to chitosan in cell walls of plant pathogenic fungi. New Phytologist.

[70] O’Connell RJ, Ride JP (1990). Chemical detection and ultrastructural localization of chitin in cell walls of Colletotrichum lindemuthianum. Physiol Mol Plant Pathology.

[71] Abelson PH (1999). A potential phosphate crisis. Science.

[72] Mullaney EJ, Daly CB, Ullah A (1999). Advances in phytase research. Adv Appl Microbiol.

[73] Tang W (2006). The application of laser microdissection to in planta gene expression profiling of the maize anthracnose stalk rot fungus Colletotrichum graminicola. Mol Plant Microbe Interact.

[74] Goodwin PH, Li J, Jin S (2000). Evidence for sulfate derepression of an arylsulfatase gene of Colletotrichum gloeosporioides f. sp. malvae during infection of round-leaved mallow, Malva pusilla. Physiol Mol Plant Pathology.

[75] Sohn J (2000). High level activation of vitamin B1 biosynthesis genes in haustoria of the rust fungus Uromyces fabae. Mol Plant Microbe Interact.

[76] Percudani R, Peracchi A (2003). A genomic overview of pyridoxal‐phosphate‐dependent enzymes. EMBO Rep.

[77] Thara VK, Fellers JP, Zhou JM (2003). In planta induced genes of Puccinia triticina. Mol Plant Pathol.

[78] Struck C (2004). The Uromyces fabae UfAAT3 gene encodes a general amino acid permease that prefers uptake of in planta scarce amino acids. Mol Plant Pathol.

[79] Bilski P (2000). Vitamin B6 (Pyridoxine) and Its Derivatives Are Efficient Singlet Oxygen Quenchers and Potential Fungal Antioxidants. Photochem Photobiol.

[80] Titiz O (2006). PDX1 is essential for vitamin B6 biosynthesis, development and stress tolerance in Arabidopsis. Plant J.

[81] DeZwaan TM (1999). Magnaporthe grisea pth11p is a novel plasma membrane protein that mediates appressorium differentiation in response to inductive substrate cues. Plant Cell.

[82] Kulkarni RD (2005). Novel G-protein-coupled receptor-like proteins in the plant pathogenic fungus Magnaporthe grisea. Genome Biol.

[83] Vaillancourt LJ, Hanau RM (1994). Nitrate-nonutilizing mutants used to study heterokaryosis and vegetative compatibility in Glomerella graminicola (Colletotrichum graminicola). Exp Mycol.

[84] Kubicek CP, Starr TL, Glass NL (2014). Plant cell wall-degrading enzymes and their secretion in plant-pathogenic fungi. Annu Rev Phytopathol.

[85] Catanzariti A-M (2006). Haustorially Expressed Secreted Proteins from Flax Rust Are Highly Enriched for Avirulence Elicitors. Plant Cell.

[86] Koeck M, Hardham AR, Dodds PN (2011). The role of effectors of biotrophic and hemibiotrophic fungi in infection. Cell Microbiol.

[87] Ökmen B, Doehlemann G (2014). Inside plant: biotrophic strategies to modulate host immunity and metabolism. Curr Opin Plant Biol.

[88] Kleemann J (2008). Identification of soluble secreted proteins from appressoria of Colletotrichum higginsianum by analysis of expressed sequence tags. Microbiology.

[89] Guyon K (2014). Secretome analysis reveals effector candidates associated with broad host range necrotrophy in the fungal plant pathogen Sclerotinia sclerotiorum. BMC Genomics.

[90] Lee S-J, Rose JKC (2010). Mediation of the transition from biotrophy to necrotrophy in hemibiotrophic plant pathogens by secreted effector proteins. Plant Signal Behav.

[91] Schulze-Lefert P, Panstruga R (2011). A molecular evolutionary concept connecting nonhost resistance, pathogen host range, and pathogen speciation. Trends Plant Sci.

[92] Pedersen C (2012). Structure and evolution of barley powdery mildew effector candidates. BMC Genomics.

[93] Dong S (2014). Effector specialization in a lineage of the Irish potato famine pathogen. Science.

[94] Mosquera G (2009). Interaction transcriptome analysis identifies Magnaporthe oryzae BAS1-4 as biotrophy-associated secreted proteins in rice blast disease. Plant Cell.

[95] Xue C (2002). Two novel fungal virulence genes specifically expressed in appressoria of the rice blast fungus. Plant Cell.

[96] Marshall R (2011). Analysis of two in planta expressed LysM effector homologs from the fungus Mycosphaerella graminicola reveals novel functional properties and varying contributions to virulence on wheat. Plant Physiol.

[97] Mentlak TA (2012). Effector-mediated suppression of chitin-triggered immunity by Magnaporthe oryzae is necessary for rice blast disease. Plant Cell.

[98] Kombrink A, Thomma BPHJ (2013). LysM effectors: secreted proteins supporting fungal life.

[99] Dong S (2012). The NLP toxin family in Phytophthora sojae includes rapidly evolving groups that lack necrosis-inducing activity. Mol Plant Microbe Interact.

[100] Ottmann C (2009). A common toxin fold mediates microbial attack and plant defense. Proc Natl Acad Sci.

[101] Fudal I (2007). Expression of Magnaporthe grisea avirulence gene ACE1 is connected to the initiation of appressorium-mediated penetration. Eukaryot Cell.

[102] Collemare J (2008). Biosynthesis of secondary metabolites in the rice blast fungus Magnaporthe grisea: the role of hybrid PKS-NRPS in pathogenicity. Mycol Res.

[103] Wang S (2008). Functional characterization of the biosynthesis of radicicol, an Hsp90 inhibitor resorcylic acid lactone from Chaetomium chiversii. Chem Biol.

[104] Zhou H (2010). Insights into radicicol biosynthesis via heterologous synthesis of intermediates and analogs. J Biol Chem.

[105] Wicklow DT, Jordan AM, Gloer JB (2009). Antifungal metabolites (monorden, monocillins I, II, III) from Colletotrichum graminicola, a systemic vascular pathogen of maize. Mycol Res.

[106] Roe SM (1999). Structural basis for inhibition of the Hsp90 molecular chaperone by the antitumor antibiotics radicicol and geldanamycin. J Med Chem.

[107] Nguyen QB (2011). Simultaneous silencing of endo‐β‐1, 4 xylanase genes reveals their roles in the virulence of Magnaporthe oryzae. Mol Microbiol.

[108] Hung C-Y (2005). A metalloproteinase of Coccidioides posadasii contributes to evasion of host detection. Infect Immun.

[109] Wu D (2012). ChLae1 and ChVel1 regulate T-toxin production, virulence, oxidative stress response, and development of the maize pathogen Cochliobolus heterostrophus. PLoS Pathog.

[110] Liebmann B (2004). Deletion of the Aspergillus fumigatus lysine biosynthesis gene lysF encoding homoaconitase leads to attenuated virulence in a low-dose mouse infection model of invasive aspergillosis. Arch Microbiol.

[111] Venditti R (2012). Sedlin controls the ER export of procollagen by regulating the Sar1 cycle. Science.

[112] Doehlemann G (2008). Reprogramming a maize plant: transcriptional and metabolic changes induced by the fungal biotroph Ustilago maydis. Plant J.

[113] van der Linde K (2011). Systemic virus‐induced gene silencing allows functional characterization of maize genes during biotrophic interaction with Ustilago maydis. New Phytol.

[114] Hückelhoven R (2004). BAX Inhibitor-1, an ancient cell death suppressor in animals and plants with prokaryotic relatives. Apoptosis.

[115] Eichmann R (2004). The barley apoptosis suppressor homologue BAX inhibitor-1 compromises nonhost penetration resistance of barley to the inappropriate pathogen Blumeria graminis f. sp. tritici. Mol Plant Microbe Interact.

[116] Van Loon LC, Rep M, Pieterse CMJ (2006). Significance of inducible defense-related proteins in infected plants. Annu Rev Phytopathol.

[117] Rakwal R, Agrawal GK, Jwa N-S (2001). Characterization of a rice (Oryza sativa L.) Bowman–Birk proteinase inhibitor: tightly light regulated induction in response to cut, jasmonic acid, ethylene and protein phosphatase 2A inhibitors. Gene.

[118] Thomma BPHJ (1998). Separate jasmonate-dependent and salicylate-dependent defense-response pathways in Arabidopsis are essential for resistance to distinct microbial pathogens. Proc Natl Acad Sci.

[119] Govrin EM, Levine A (2000). The hypersensitive response facilitates plant infection by the necrotrophic pathogen Botrytis cinerea. Curr Biol.

[120] Metz RP (2006). Differential transcriptional regulation by mouse single-minded 2 s. J Biol Chem.

[121] Mortazavi A (2008). Mapping and quantifying mammalian transcriptomes by RNA-Seq. Nat Methods.

[122] Dillies M-A (2013). A comprehensive evaluation of normalization methods for Illumina high-throughput RNA sequencing data analysis. Brief Bioinform.

[123] Robinson MD, Oshlack A (2010). A scaling normalization method for differential expression analysis of RNA-seq data. Genome Biol.

[124] Benjamini Y, Hochberg Y (1995). Controlling the False Discovery Rate: A Practical and Powerful Approach to Multiple Testing. J R Stat Soc Series B.

[125] Sturn A, Quackenbush J, Trajanoski Z (2002). Genesis: cluster analysis of microarray data. Bioinformatics.

[126] Pfaffl MW (2001). A new mathematical model for relative quantification in real-time RT–PCR. Nucleic Acids Res.

[127] Blüthgen N (2005). Biological Profiling of Gene Groups utilizing Gene Ontology. Genome Inform.

[128] Horton P (2007). WoLF PSORT: protein localization predictor. Nucleic Acids Res.

[129] Punta M, et al. The Pfam protein families database. Nucleic acids research, 2011. doi:10.1093/nar/gkr1065.

[130] Saier MH, Tran CV, Barabote RD (2006). TCDB: the Transporter Classification Database for membrane transport protein analyses and information. Nucleic Acids Res.

[131] Cantarel BL (2009). The Carbohydrate-Active EnZymes database (CAZy): an expert resource for Glycogenomics. Nucleic Acids Res.

[132] Yin Y (2012). dbCAN: a web resource for automated carbohydrate-active enzyme annotation. Nucleic Acids Res.

[133] Rawlings ND, Barrett AJ, Bateman A (2010). MEROPS: the peptidase database. Nucleic Acids Res.

