# Supplementary material for: A Colletotrichum graminicola mutant deficient in the establishment of biotrophy reveals early transcriptional events in the maize anthracnose disease interaction
Source: BMC Genomics. 2016 Mar 8;17:202. doi: 10.1186/s12864-016-2546-0 (PMC4782317; doi:10.1186/s12864-016-2546-0)

**Supplemental Figures**

**Figure S1.** Phenotypes of WT (A-C) and MT (D-E) on maize leaf sheaths. Pre-penetration appressoria (A and D) samples were collected 18-24 hpi. Biotrophic samples (B and E) were collected 36-48 hpi. Necrotrophic samples (C) were collected 60-65 hpi. AP= Appressoria. BH=Biotrophic hyphae. NH= Necrotrophic hyphae. Note that the numbers of appressoria produced by the MT and WT strains is similar at 24 hpi (Torres et al., 2014), even though panel A shows more appressoria than panel B.


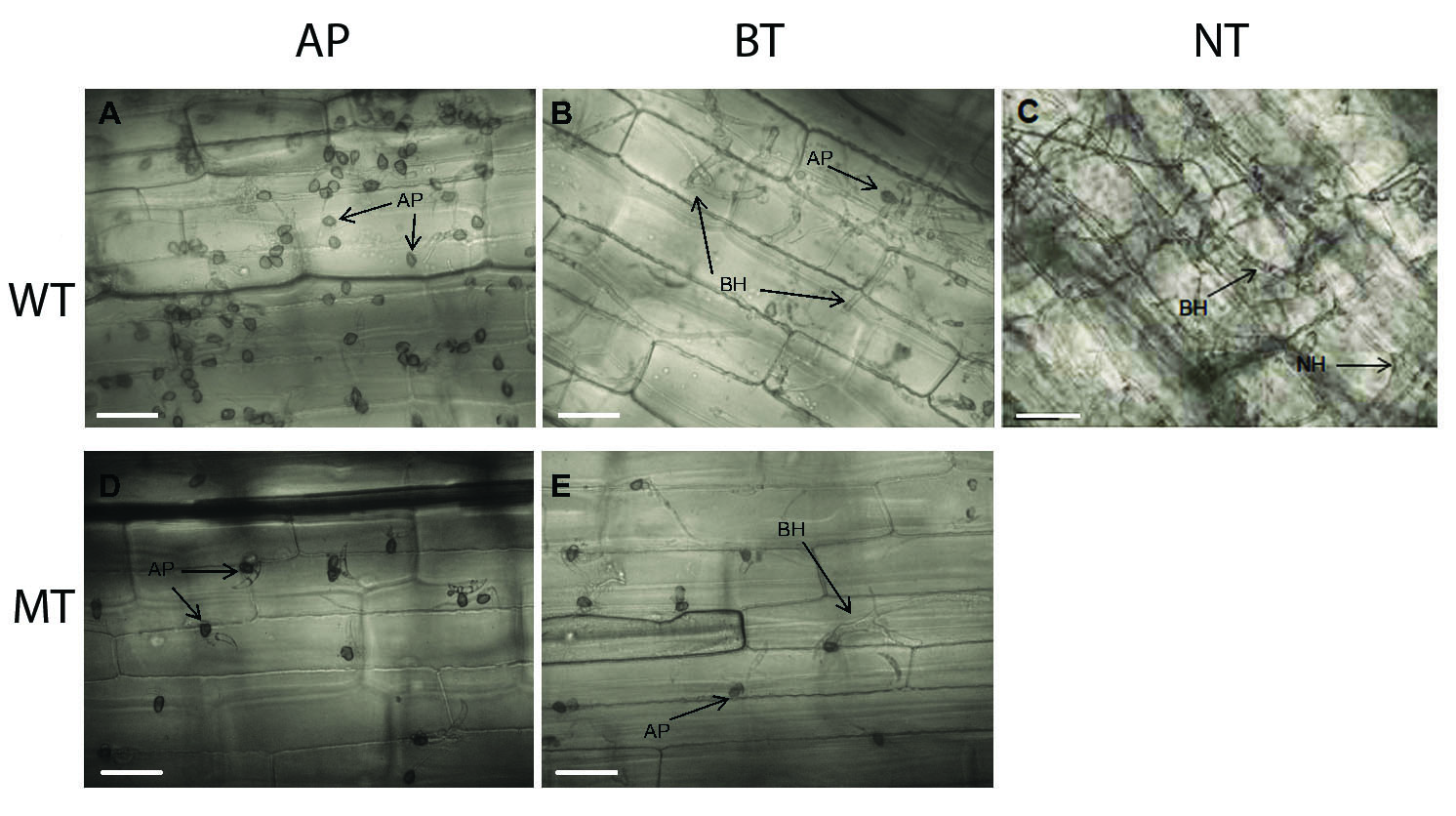


**Figure S2**: Correlation analysis of gene expression profiles generated by RNA-Seq and qRT-PCR for 38 individual comparisons involving fourteen fungal genes.

**Figure S3.** Quantitative RT-PCR of putative secreted effector protein genes. BAS2 and BAS3= biotrophy-associated proteins 2 and 3. Expression in different stages of fungal infection in the WT (black bars), *Cpr1*-C (white bars) and *cpr1* mutant (dark gray bars). Expression values are shown as fold changes relative to expression in other fungal stages. IVAP= *in vitro* appressoria. AP= appressoria . BT= biotrophic stage NT= necrotrophic stage.


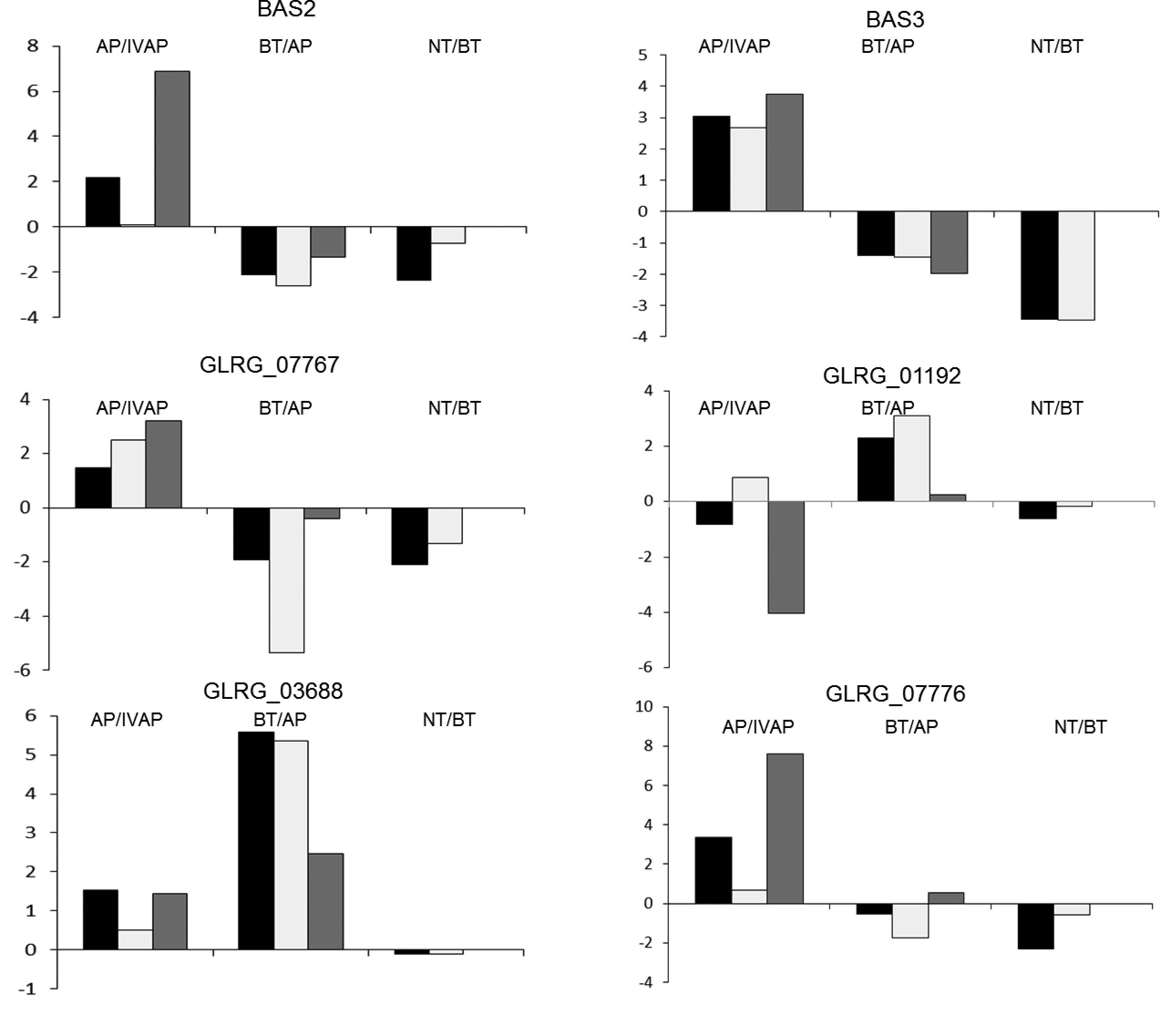


**Figure S4**. Quantitative RT-PCR of genes from clusters 18, 22 and 35 in WT (black bars), *Cpr1*-C (white bars) and *cpr1* mutant (dark gray bars). Expression values are shown as fold changes relative to expression in other fungal stages. IVAP= *in vitro* appressoria. AP= appressoria . BT= biotrophic stage NT= necrotrophic stage.


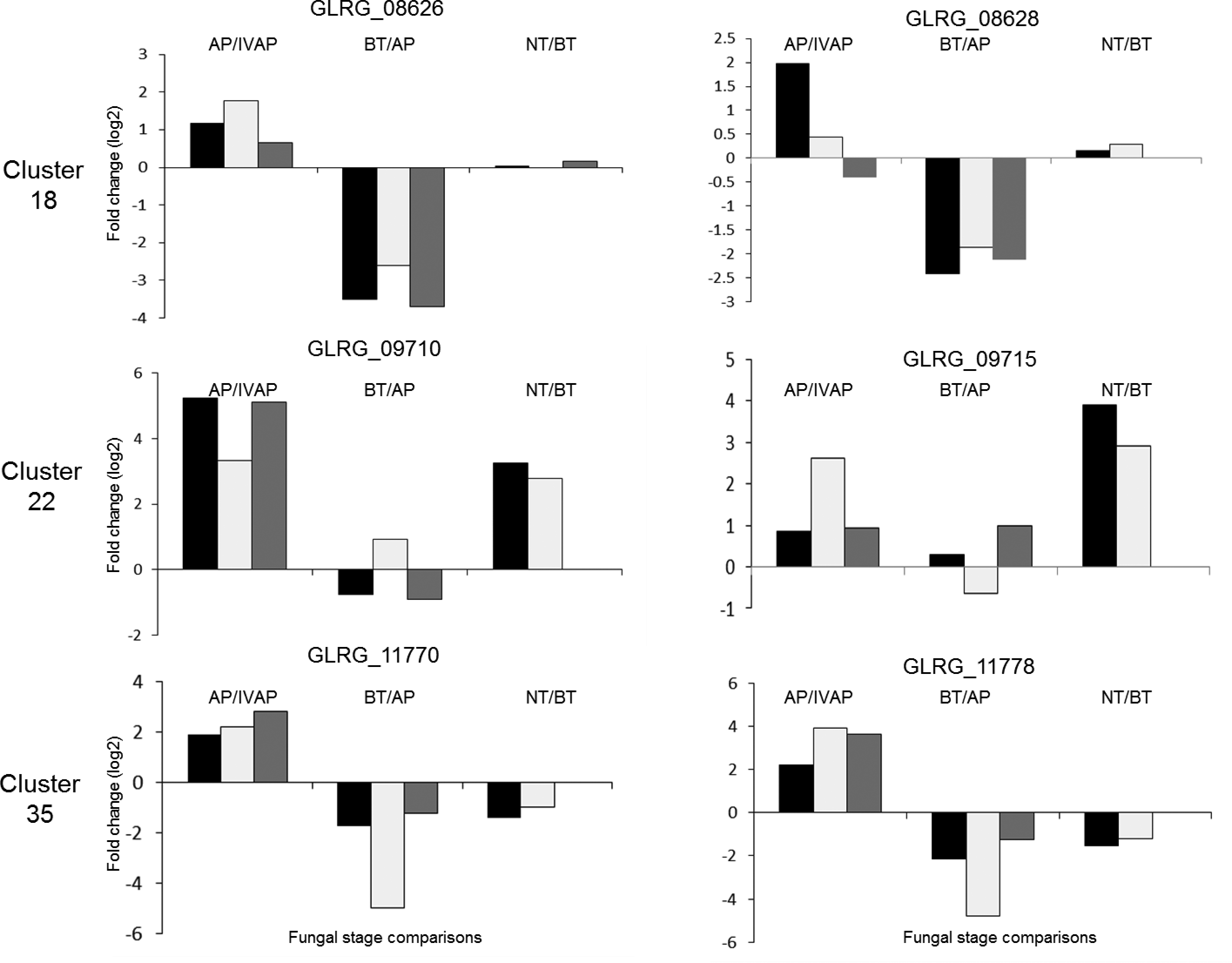

Supplement: Additional file 1: Figure S1. — Supplemental Figures. (DOCX 13389 kb) [file 12864_2016_2546_MOESM1_ESM.docx]
